# Mild Warming Induces Divergent Plastic Responses in Gene Expression Among Populations of a Temperate Butterfly

**DOI:** 10.1111/mec.70309

**Published:** 2026-04-01

**Authors:** N. Verspagen, M. Frapin, R. Das Roy, H. Mäkinen, M. Saastamoinen, M. F. DiLeo

**Affiliations:** ^1^ Department of Organismal and Evolutionary Biology, Faculty of Biological and Environmental Sciences University of Helsinki Helsinki Finland; ^2^ Helsinki Institute of Life Science University of Helsinki Helsinki Finland; ^3^ Biodata Analytics Unit, Institute of Biotechnology University of Helsinki Helsinki Finland; ^4^ Wildlife Research and Monitoring Section Ontario Ministry of Natural Resources Peterborough Ontario Canada

**Keywords:** environmental gradient, insect, intraspecific variation, phenotypic plasticity, RNAseq, transcriptome

## Abstract

In response to potentially stressful conditions, for example due to climate change, organisms can move, adjust through phenotypic plasticity and evolve. Evolution can act on mean trait values, or on plasticity itself. Whether phenotypic plasticity, evolution, or evolution of plasticity is the dominant response to environmental change is still subject to debate. Representing a close link between genotype and phenotype, gene expression studies are an effective tool to study these questions, especially combined with environmental clines. Therefore, we used a common garden experiment to quantify gene expression across two populations of Glanville fritillary butterflies originating from different latitudes in Europe, tested at two temperature treatments. We investigated gene expression patterns using differential expression analysis and weighted gene co‐expression network analysis (WGCNA) to disentangle genetic from plastic effects. We found that, regardless of population of origin, most differentially expressed genes and co‐expression modules were responsive to temperature. We also found strong evidence for variation in plasticity between populations (GxE) in both gene‐by‐gene and co‐expression analyses, but only little evidence for genetic assimilation or compensation. While a large proportion of differentially expressed genes showed a reaction norm reversal, this was not the case for co‐expression modules. This indicates the importance of considering coordinated, low‐level changes across many genes. Biological processes overrepresented in genes and gene co‐expression modules differentially expressed with temperature and/or the population by temperature interaction were generally related to cellular maintenance and growth. Our results highlight the importance of intraspecific differences in phenotypic plasticity in response to mild warming.

## 1 Introduction

As global temperatures rise due to climate change, extreme events such as heatwaves are becoming more frequent (Easterling et al. 2000; Meehl and Tebaldi 2004; Ragone et al. 2018; Russo et al. 2015). This may lead to conditions transcending organisms' thermal niches more frequently (Gvoždík 2018; McMahon and Hays 2006) and insects, being ectotherms, may be especially vulnerable to these changes (Angilletta Jr 2009; Stange and Ayres 2010). In response to such potentially stressful changing climatic conditions, some organisms adjust their range and move to more suitable conditions (McCain and Garfinkel 2021; Parmesan and Yohe 2003). However, this possibility is limited, for example, by resource distribution or dispersal ability. Organisms can also adjust to the new conditions in situ through phenotypic plasticity, or adapt through evolution (Beldade et al. 2011; Hoffmann and Sgrò 2011). Phenotypic plasticity offers a fast response to new conditions, but may be limited (Gunderson and Stillman 2015), for example due to costs or limits of plasticity (Auld et al. 2009; DeWitt et al. 1998). Furthermore, the effectiveness of phenotypic plasticity depends on environmental predictability of future conditions (Hoffmann and Bridle 2022; Vinton et al. 2022). Besides plasticity, evolution might be needed for populations to survive changing conditions, especially across longer timescales (Bell 2017). Evolution can act on mean trait values, leading to local adaptation, but can also act on plasticity itself, either increasing or decreasing responsiveness to environmental conditions (Scheiner 1993; Via and Lande 1985). Whether plasticity, evolution, or evolution of plasticity, in the form of significant interactions between genetic background and environmental response (GxE), is the dominant response to changing environmental conditions is still subject to debate (Kelly 2019). To accurately predict species' responses to future climate change, more information is needed on the interactions between evolution and phenotypic plasticity.

To study such interactions, environmental gradients in combination with common garden or translocation experiments are powerful tools, as they allow disentangling genetic from plastic responses. Here, space‐for‐time substitutions can be used to estimate responses to future warming. Populations from warmer regions (i.e., lower altitudes or latitudes) are used as proxies for future warming and considered as heat‐tolerant, as compared to populations from colder regions (i.e., higher altitudes or latitudes) which are considered heat‐sensitive (De Frenne et al. 2013; Merilä and Hendry 2014; Verheyen et al. 2019). Gene expression represents a close link between the genome and the phenotype, and as every gene can be considered a molecular phenotype, sequencing of gene expression levels through RNAseq provides a large dataset of multiple traits at a given time (Chevin et al. 2022; DeBiasse and Kelly 2016). Combined with common garden experiments, gene expression studies are thus a powerful tool to investigate the mechanisms underlying the impact of environmental conditions on life‐history (DeBiasse and Kelly 2016; Stanford et al. 2020; Swaegers and Koch 2022; Todd et al. 2016), especially together with functional annotation of genes, or associated with measured phenotypic traits (Ashburner et al. 2000; Langfelder and Horvath 2008). Gene expression studies have traditionally been performed by assessing expression differences for each gene, but outcomes of these studies are heavily dependent on significance thresholds (Swaegers and Koch 2022). Furthermore, single genes do not act in isolation but are rather part of functionally similar gene networks (Davidson 2010). The use of Weighted Gene Co‐expression Network Analysis (WGNA) reduces the problem of multiple testing associated with single gene analyses, and better detects coordinated, low‐level changes across many genes (Langfelder and Horvath 2008; Mäkinen et al. 2018; Orsini et al. 2018; Stanford and Rogers 2018). This complements the detailed picture of gene expression obtained with more traditional gene‐by‐bene differential expression analysis.

Results from previous research using gene expression to study the interplay between evolution and phenotypic plasticity are varied. Firstly, plastic responses to temperature are generally important, where genes related to thermal stress such as heat shock proteins or oxidative stress repair are upregulated with temperature (e.g., Pimsler et al. 2020; Fangue et al. 2006; Winters et al. 2011; Franssen et al. 2014). When selection acts on such plasticity, it may lead to evolution of plasticity in the forms of GxE interactions. For example, in *Drosophila*, heat‐tolerant populations were more plastic than heat‐sensitive populations (Levine et al. 2011) indicating that phenotypic plasticity was beneficial. However, if the initial plastic response to temperature is beneficial, it may also become fixed in the population more regularly exposed to warm conditions, leading to a loss of plasticity through the process of genetic assimilation (Loison 2019; Pigliucci et al. 2006). Patterns of genetic assimilation in gene expression have been found for example in common killifish (Fangue et al. 2006), corals (Barshis et al. 2013; Kenkel et al. 2013), and seagrass (Franssen et al. 2014). Alternatively, when the initial plastic response to temperature is maladaptive, it may be lost in the heat‐tolerant population, leading to genetic compensation (Grether 2005). Here, expression is upregulated with temperature in heat‐sensitive populations but remains at low levels in heat‐tolerant populations, as in the damselfly *Ischnura elegans* (Swaegers et al. 2020). GxE responses in the literature are thus mixed, but much of this literature is focused on response to acute thermal stress, while less attention has been paid to chronic exposure of mild increased temperatures. This is especially the case in terrestrial insects. Therefore, more research on species with different ecological backgrounds and under different thermal conditions is needed to explore which factors affect the evolution of phenotypic plasticity and how species may respond to future climate warming.

In this study, we used a space‐for‐time approach to analyse the interaction between phenotypic plasticity and evolution using RNAseq in two populations of Glanville fritillary butterfly (*Melitaea cinxia*) across a latitudinal gradient (Finland and Spain). The northern, Finnish population, was used as a proxy for the heat‐sensitive population in the face of climate change, while the southern, Spanish population, which originates from a warmer environment, was used as a proxy for the future heat‐tolerant population. We integrated this sampling design with a reactionome, the gene expression reaction norm for all genes across an experimental gradient, (Stanton‐Geddes et al. 2016) as outlined by Swaegers and Koch (2022). To better understand responses of all genes separately as well as coordinated changes among gene networks, we combined gene‐by‐gene differential expression analysis with WGCNA analysis. The question we aimed to answer was: is evolution (G), plasticity (E), or evolution of plasticity (GxE) the most dominant response to chronic mild thermal stress? We paid special attention to signatures of genetic assimilation or compensation to understand if plasticity is adaptive or not. If gene expression differs between test temperatures but not between populations, plasticity might be an important and adequate response to temperature, eliminating the need for evolution. Similarly, if we find divergence in gene expression patterns between populations but no interactions with temperature, this might indicate that evolution in response to differences in thermal conditions between the two populations has occurred while plasticity is of less importance. Finally, if we find shifts in reaction norm slopes as the most common difference between populations (i.e., GxE), evolving plasticity might be an important part of the adaptive response to warming temperatures. We also aimed to explore the biological processes that are overrepresented in any differentially expressed genes, as these can shed light on which traits are mostly impacted by climate change.

## 2 Methods

### 2.1 Data Background

For a full background on experimental populations and design used in this study, see Verspagen et al. (2023). In short, the Glanville fritillary butterflies (*Melitaea cinxia*) used in this experiment originated from two locations at opposite ends of their latitudinal range, Finland and Spain (see Table S1 for detailed collection locations). Although bivoltine populations exist, the populations used here are strictly univoltine. Females lay egg clutches of 100–200 eggs (Saastamoinen 2007). The F0 generation from Spain was collected as egg clutches directly from 10 adults in spring 2019 and reared gregariously under standard conditions (28°C during the day and 15°C during the night, with L12:D12 h day: night conditions) until diapause. The F0 generation from Finland was collected in autumn 2019 as individual diapausing larvae from 22 different families. In autumn 2019, larvae from both populations were transferred to diapause conditions (constant darkness at 5°C and 80% relative humidity), as winter diapause during the larval stage is obligatory in these populations of the butterfly.

In February 2020, after roughly six months of diapause, rearing was continued for all larvae at standard rearing conditions. Larvae were fed daily with leaves of host plant *Plantago lanceolata* , *ad libitum*. Upon eclosion, butterflies were marked, females were mated with males from the same region but from a different family, and the offspring of 11 females (hereafter called family) per population (F1 generation) were used in this experiment. When resulting egg clutches hatched and larvae moulted to the second instar, 15 individuals from each family were weighed and placed at different thermal treatments with four daytime temperatures (25°C, 28°C, 31°C and 34°C, reflecting mild warming), each with a constant night temperature of 8°C and a 12:12 light: dark cycle, with two replicate groups for each family per temperature. The lowest temperature treatment (25°C–8°C) reflects a rough estimate of average temperatures in the months when the pre‐diapause larvae are estimated to be developing in Finland, where mean temperature at collecting locations in July and August is 17.4°C (Copernicus Climate Change Service 2022). The upper range of our temperature treatments (34°C–8°C) is slightly elevated compared to an estimate of experienced temperatures for developing larvae in Spain, with a mean temperature at collecting locations in June and July of 19.6°C (Copernicus Climate Change Service 2022). Overall, daytime temperatures reflect conditions that can be experienced by larvae in the field, as microclimate temperature can be up to 20°C higher than ambient temperatures in sunny spots, where larval nests tend to be (Bennett et al. 2015; Rytteri et al. 2021). To make sure that all sampled larvae were in the same instar, we randomly selected three individuals from each experimental group for RNA sequencing 3 days after the group (i.e., more than 50% of the larvae) moulted to the third larval instar. These individuals were picked from the petri‐dish and immediately flash‐frozen in liquid nitrogen, and stored at −80°C. The remaining larvae were reared until diapause, upon which individual mass, development time and growth rate were determined, and these phenotypic data were used in Verspagen et al. (2023).

For the present study, due to logistical reasons, only two populations (Finland and Spain), two temperatures (25°C and 34°C), and a subset of the experimental families and treatments were used. A low number of biological replicates has been a problem in previous RNAseq studies (Auer and Doerge 2010; Soneson and Delorenzi 2013; Todd et al. 2016), and thus we decided to increase the number of biological replicates at the cost of a reduced number of study populations and temperatures. Although testing several populations along a latitudinal cline, at several temperatures, would be desirable, we are confident that outcomes from this study reflect a true latitudinal and temperature cline due to the phenotypic results we previously obtained (Verspagen et al. 2023). Because our research questions focus on reaction norm responses across populations instead of intra‐population variability, we chose to use the four families per population with the lowest within‐family variability in integral growth rate. Since intrapopulation variance is often high in natural populations (Granados‐Cifuentes et al. 2013) and has also been shown to be high in Glanville fritillary butterfly populations from Finland (Kahilainen et al. 2022; Verspagen et al. 2020), this approach increased detectability of differences between populations. We selected four families from each population with the lowest standard deviation in growth rate at 34°C (Figure S1), the temperature where variation was highest. We furthermore made sure that growth rate reaction norm slopes of the chosen families were intermediate to reflect the average response. In the Spanish population, one of the four families with the lowest standard deviation also had the lowest reaction norm slope. Therefore, we instead used the family with the fifth‐lowest standard deviation. This setup produced a full factorial split‐brood design with two populations, four families per population, two temperatures, two replicates per family in each temperature and three individuals per replicate, resulting in a final sample size of 96 individuals.

### 2.2 DNA and RNA Extraction and Sex Determination

Total RNA and DNA from whole larvae were extracted simultaneously with the Nucleospin RNA kit (Macherey‐Nagel) combined with the Nucleospin RNA/DNA buffer set (Macherey‐Nagel). Frozen larvae were transferred to a tube containing 350 μL lysis buffer RA1, 3.5 μL β‐mercaptoethanol and a stainless‐steel bead of 5 mm (QIAGEN) and homogenised using the QIAGEN TissueLyzer II for two times 30 s. The following steps were carried out according to the manufacturer's protocol: DNA was eluted in 100 μL of DNA elute and total RNA was eluted in 60 μL of RNase‐free H_2_O. Nucleic acid quantity and integrity were assessed with the NanoDrop ND‐1000 spectrophotometer (Thermo scientific) and on a 2% agarose gel.

As sex cannot be determined from the phenotype in pre‐diapause larvae, we performed sex determination of larvae via genotyping to be able to control for sex in differential expression analysis. As described in the Supporting Information for Kahilainen et al. (2022), only Lepidoptera males should be heterozygous for SNPs on the Z‐chromosome. We therefore determined the sex of the larvae by genotyping specific polymorphisms found in Glanville fritillary butterflies from Finland and/or Spain and located on this chromosome using multiplex PCR. The multiplex PCR was conducted using PhusionTM Plus DNA Polymerase (ThermoFisher Scientific) in a final volume of 10 μL. To avoid primer dimers, primers were split into two different pools with equal amounts of forward and reverse strands and at a final concentration between 0.1 and 0.2 μM. 5–50 ng of DNA was used per reaction. The PCR was carried out according to the manufacturer's protocol with 30 cycles and the PCR products for the same samples obtained with the two different pools were then combined. The following steps were carried out by the DNA Sequencing and Genomics Laboratory (BIDGEN) at the Institute of Biotechnology, University of Helsinki: briefly, multiplex PCR products were purified with ExoSAP‐IT (Thermo Fisher Scientific). Subsequently, a second PCR, using Phusion HS II Polymerase, was carried out to add on the dual indexes. Then additional purification steps were performed on the pooled PCR products before sequencing using MiSeq (Illumina). The FastQ files obtained were trimmed with Trimmomatic (version 0.39, Bolger et al. 2014) then aligned with BWA (version 0.7.17, Li and Durbin 2009). Target SNP variant calling was done with BCFtools (version 1.17, Danecek et al. 2021) and read depths were counted with HTseq‐counts (version 2.0.3, Anders et al. 2015). The sex determination of the larvae used in this study was based on 21 SNPs and 12 SNPs for the Finnish and Spanish populations respectively (Table S2).

### 2.3 RNAseq Data Analysis and WGCNA

Total RNA was sent to BGI‐Hong Kong for RNA‐sequencing. Sequencing was performed on the DNBseq platform from strand‐specific mRNA libraries, generating 100 bp paired‐end reads. The RNAseq reads were processed and aligned to the Glanville fritillary butterfly reference genome according to the nf‐core/rnaseq (version 3.11.2) pipeline of the nf‐core collection of workflows (Ewels et al. 2020). In summary, the quality of RNAseq reads were evaluated using FastQC (version 0.11.9, Andrews 2010), adapter sequences were filtered out with Cutadapt (version 3.4, Martin 2011), and ribosomal RNA was removed using Sortmerna (version 4.3.4, Kopylova et al. 2012). The average number of quality filtered reads was 24 M (standard deviation 0.48) per individual. The reads were aligned to the Glanville fritillary butterfly reference genome (PubMed: 35022701) using Salmon (version 1.10.1, Patro et al. 2017). For data exploration, we normalised the count data using the rlog transformation in the DeSeq2 package (version 1.36.0, Love et al. 2014). The rlog method provided a smoother mean count to standard deviation ratio in gene counts than the variance stabilising normalisation (Figure S2). After rlog normalisation, we removed one outlier individual (Finland, 34°C) from the data based on its position in the hierarchical clustering dendrogram (Figure S3). We then performed an exploratory PCA on the 2000 highest expressed genes, and PC 1 and 3 together separated temperature and population and, to a lesser extent, family and biological replicate. Sexes did not group clearly (Figure S4). PC2 was omitted because it formed a horseshoe pattern together with PC 1, indicating an unknown gradient in our data unexplained by phenotypic data or experimental design, and did not separate any of the experimental groups.

Differential expression analysis was performed on raw gene count data that was normalised for library size using DESeq2, after genes with low expression (count of < 5 in more than 80 samples) were filtered out. We then performed a negative binomial mixed effect model with GlmmSeq (Lewis et al. 2022) using the glmmTMB method. We included the main effects of temperature, population and sex and the interaction between temperature and population. Family was included as a random effect. We then calculated log_2_ fold change for temperature by using the model predictions, subtracting the 25°C count estimated mean (averaged over sex and population) from that of the 34°C treatment. For population, log_2_ fold change was calculated by subtracting predicted mean averaged over sex and temperature of Finland from that of Spain. Log_2_ fold change for the interaction was calculated through the formula (mean Spain 34—mean Spain 25)—(mean Finland 34—mean Finland 25). We then selected the significant up‐ or downregulated genes for each model (FDR adjusted *q* value < 0.05) that also had an absolute log2 fold change value higher than one. Post hoc testing was done though the emmeans package (Lenth et al. 2025) on all genes significant for at least both temperature and the temperature‐by‐population interaction to determine patterns of assimilation or compensation. Similarly to Harry‐Paul et al. (2024), we classified genes as ‘assimilation’ when there was a significant difference between 25°C and 34°C in the Finnish (heat‐sensitive) but not in the Spanish (heat‐tolerant) population, and the two populations were significantly different from each other at 25 but not at 34°C. We classified genes as ‘compensation’ when there was a significant difference between 25°C and 34°C in the Finnish (heat‐sensitive) but not in the Spanish (heat‐tolerant) population, and the two populations were significantly different from each other at 34 but not 25°C.

Next, we used WGCNA to detect functionally related gene expression modules. WGCNA was performed on non‐filtered rlog transformed genes, and we removed 313 genes with zero variance. We constructed a gene co‐expression network by first choosing a soft thresholding power estimated from the data using the pickSoftThreshold function in the WGCNA R package (version 1.71, Langfelder and Horvath 2008). The purpose of the soft threshold is to raise each pairwise gene correlation to a power that meets the scale free network topology assumption. As recommended by the WGCNA manuals, we chose a soft threshold value (4) where the R2 value is higher than 0.8, the correlation curve as function of soft threshold starts to plateau (Figure S5A) and the mean connectivity (sum of correlation coefficients) is higher than 100 (Figure S5B). We constructed a signed gene co‐expression network using the blockwiseModules function using a soft threshold power of four. The detection of expression modules was based on hierarchical clustering and dynamic tree cut method with parameters: minimum module size 50, merge cut height 0.1 and deep split 4. As a result, genes were assigned to modules except those genes not meeting the dynamic tree cut thresholds (Figure S6). Seventeen co‐expressed modules were detected in the WGCNA analysis. Modules that were closely correlated to each other (i.e., eigengene expression had a Pearson's correlation coefficient > 0.75) were merged, resulting in eleven co‐expressed modules (Figure S6). As a representative of gene expression in each module, eigengenes (PC1) were calculated as implemented in the WGCNA R package.

After initial exploratory analysis, some module eigengenes seemed to be bimodally distributed and this was unexplained by population, temperature, or sex. Therefore, we calculated bimodality indices for the residuals of a linear model of eigengene ~ population + temperature + sex using the BimodalIndex package (Coombes 2019). Five modules (Modules 1, 8, 9, 10 and 11) had a bimodal index higher than one and were classified as bimodal (Figure S7). We analysed the effects of temperature, population, their interaction and sex on module eigengene expression using quantile regression with the lqmm package (Geraci and Bottai 2014). Family was included as a random effect. We analysed the 0.5 quantile, and additionally the 0.25 and 0.75 quantiles for bimodal modules. We then performed post hoc testing to detect assimilation or compensation on the modules that were significantly differentially expressed for both temperature and population following a similar procedure as the gene‐by‐gene analysis. Since post hoc testing could only be performed on the 0.5 quantile, we excluded all bimodal modules from this analysis.

We finally performed gene ontology (GO) term overrepresentation analysis both on the individual differentially expressed genes for temperature, population of origin and their interaction, and on the co‐expression modules. *p* values were adjusted for multiple testing using the Benjamini–Hochberg method and we used adjusted *p*‐value cutoff of 0.05. We used semantic similarity to cluster similar GO terms. Analysis, cluster simplification and visualisation were performed with clusterProfiler (version 4.4.4, Yu et al. 2012) and enrichplot (version 1.16.1, Yu et al. 2023). The reference genome, gene annotation and GO term annotations were downloaded from the genome assembly of the Glanville fritillary butterfly (Smolander et al. 2022).

## 3 Results

### 3.1 Differential Expression Analysis

**FIGURE 1:**
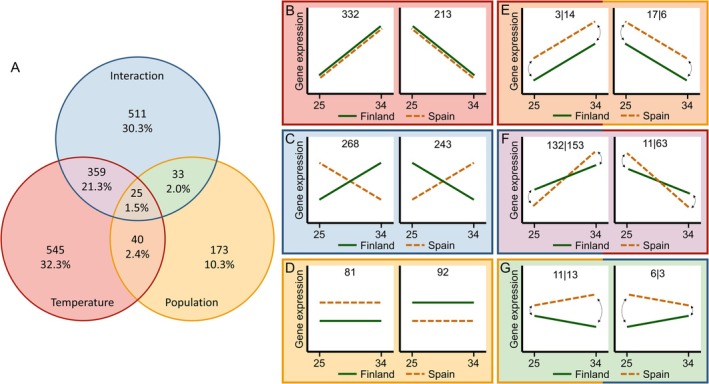
(A) Venn diagram of differential expression for temperature (red), population (yellow) and their interaction (blue). Panels B‐G show a depiction of the theoretical reaction norm for each category with the number of genes within each category. (B) Reaction norm for the main effect of temperature. Genes can be upregulated (left) or downregulated (right) with an increase in temperature, but slope and intercept are the same for the two populations. (C) Reaction norm for the population by temperature interaction without main effects of temperature or population, resulting in crossing reaction norms. (D) Reaction norm for the main effect of population, where the reaction norm is flat due to the lack of temperature or interaction effects but genes are either upregulated in Spain (left) or in Finland (right). (E) Reaction norms for the overlapping effects of temperature and population. Expression can be upregulated (left) or downregulated (right) with increasing temperature, as well as higher in Spain (shown, numbers left of vertical line) or in Finland (arrows, numbers right of vertical line). (F) Reaction norms for the overlapping effects of temperature and the interaction between temperature and population as indicated by upregulation (left) or downregulation (right) with temperature and a difference in reaction norm slopes between populations, with increased temperature response in Spain (shown, numbers left of vertical line) or Finland (arrows, numbers right of vertical line). (G) Reaction norms for the overlapping effects of population and the interaction between temperature and population, showing upregulation in Spain (shown, numbers left of vertical line) or Finland (arrows, numbers right of vertical line) and different slopes between populations.

From a total of 14,810 genes, 5513 were filtered out due to low expression (count of < 5 in more than 80 samples). Six genes (0.04%) were differentially expressed (FDR adjusted *q* value < 0.05 and absolute log_2_ fold change > 1) due to sex. 1686 genes (11.4%) were significantly differentially expressed due to temperature, population and/or their interaction. Out of the total number of differentially expressed genes, 32.3% responded only to temperature (Figure 1A), with 12.6% downregulated and 19.7% upregulated with increasing temperature (Figure 1B). 10.3% (Figure 1A) of genes were differentially expressed due to the main effect of population, of which roughly the same number of genes had higher expression in Finland (5.5%) or Spain (4.8%, Figure 1D). 30.3% of differentially expressed genes were significant only for the interaction between temperature and population (Figure 1A). Due to the lack of significant main terms, reaction norms for the two populations crossed and temperature response in Finland was opposite to that in Spain (Figure 1C). A slightly higher proportion of genes were upregulated in the Spanish and downregulated in the Finnish population (15.9%) than vice versa (14.4%).

Generally, less genes were significant for at least two model terms. Firstly, 2.4% of differentially expressed genes differed both between the two temperatures and between populations (Figure 1A,E): 1.0% was upregulated with temperature, with 0.2% having higher expression in Spain and 0.8% having higher expression in Finland. 1.4% of differentially expressed genes was downregulated, with 1.0% having higher expression in Spain and 0.4% having higher expression in Finland. In 21.3% of differentially expressed genes, expression was significantly up‐ or downregulated with increasing temperature in both populations, but the extent of this temperature response differed (i.e., significant main effect of temperature as well as interaction, Figure 1A,F). Here, most genes were upregulated with temperature (16.9%), with Spain being more responsive in 7.8% and Finland in 9.1% of differentially expressed genes. For genes that were downregulated with temperature (4.4%), Spain was more responsive in 0.7% of genes and Finland was more responsive in 3.7% of genes. 2.0% of differentially expressed genes had significant interaction and population effects (Figure 1A), where no main effect of temperature is present but both populations have different means as well as reaction norm slopes (Figure 1A,G). Here, expression was higher and upregulated with temperature in Spain in 0.7%, and higher and downregulated with temperature in Spain in 0.4% of differentially expressed genes. Expression was higher and upregulated with temperature in Finland in 0.8% and higher and downregulated with temperature in Finland in 0.2% of differentially expressed genes. Only 1.5% of differentially expressed genes were significantly differentially expressed for temperature, population and their interaction (Figure 1A, theoretical reaction norms not shown). Further post hoc analysis on genes significant for at least temperature and the temperature by population interaction revealed 25 (1.5%) genes that showed patterns of genetic assimilation and 41 (2.4%) with patterns of genetic compensation.

### 3.2 WGCNA Analysis

**FIGURE 2:**
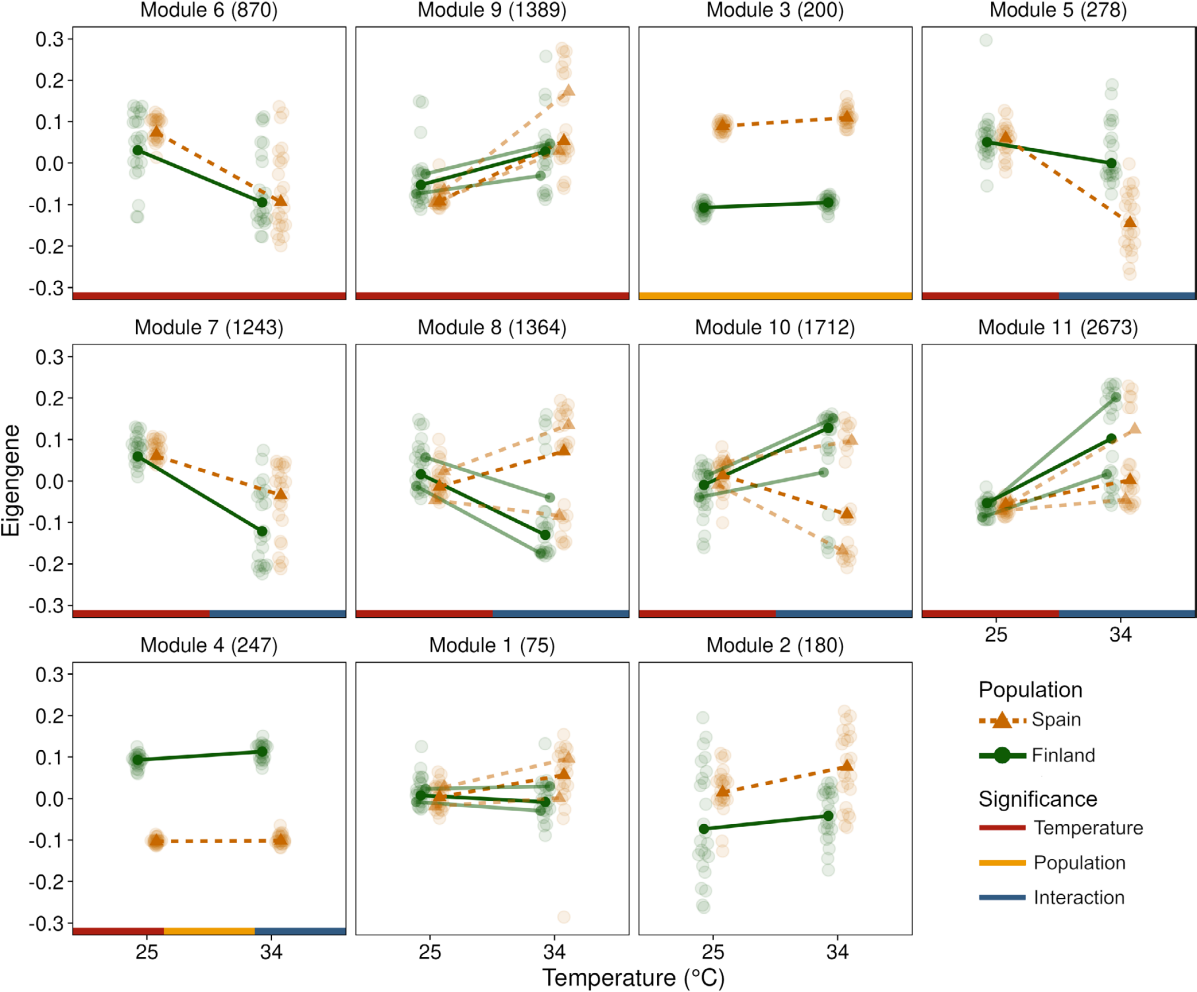
Eigengene expression for all co‐expression modules for the Finnish (dark green circles and solid line) and Spanish (brown triangles and dashed line) populations. Transparent points in the background indicate raw eigengene expression for each sample and solid points in the foreground connected by lines show mean predicted values for the 0.5 quantile. In bimodally distributed modules, semi‐transparent points in the foreground connected by lines show mean predicted values for the 0.25 and 0.75 quantiles. Coloured bands at the bottom of the panel correspond to Venn diagram colours of Figure 1 and show significance of temperature (red), population (yellow) and their interaction (blue). Four eigengenes from the module 1 with values below −0.3 were omitted from the plot to increase readability. Numbers next to the module labels show the number of genes in the module.

Of the total 14,497 genes in the data set that were left after filtering genes with zero variance, 10,231 genes were assigned to 11 modules and 4266 genes were unassigned. The number of genes assigned to each module varied substantially, with the smallest module 1 consisting of 75 genes while the largest module 11 had 2673 genes (Figure 2). Eigengene expression in five modules (1, 8, 9, 10 and 11, Figure 2) had a bimodal distribution unexplained by temperature, population or sex. This bimodal distribution was mainly present at 34°C, indicating a switch‐like response to increased temperature. The gene expression of all but modules 1 and 2 (Figure 2, Table S3) was affected by temperature, population, or their interaction. Eight out of 11 modules were affected by increasing temperature (Figure 2, Table S3), showing that phenotypic plasticity is a dominant response to warming. Eigengene expression significantly increased with an increase in temperature in four of these modules (modules 4, 9, 10 and 11) while it decreased in four other modules (5, 6, 7 and 8).

Modules 6 and 9 were only significantly differentially expressed with temperature, while the other modules (5, 7, 8, 10 and 11) were also significant for the population by temperature interaction or both the population and temperature by population interaction (module 4, although effect sizes for temperature and interaction are very small). In those modules, eigengene expression in the Finnish population responded significantly differently to temperature than that in the Spanish population, as can be seen from different reaction norm slopes. In unimodally distributed modules, the Finnish population was more responsive to temperature in two modules (4 and 7), while the Spanish population was more responsive to temperature in module 5. Three modules significant for the temperature by population interaction had a bimodal eigengene distribution. The significant interaction term in this case most likely represents differences in frequency of both modes between the populations, as can be seen from the quantile regression (Figure 2, Table S3). In module 8, eigengene expression was more frequently upregulated with temperature in the Spanish population, while it was more frequently downregulated with temperature in the Finnish population. Modules 9 and 11 show an opposite pattern. No co‐expression modules fit the criteria for assimilation or compensation, although only unimodal modules could be included in this analysis.

Eigengenes from two modules (3 and 4) were differentially expressed between the two populations. In module 3, expression increased in the Spanish population while it increased in the Finnish population in module 4 (Figure 2, Table S3). Finally, two modules (7 and 11) were significant for sex, but effect sizes were small (Figure S8, Table S3).

### 3.3 Overrepresentation Analysis

**FIGURE 3:**
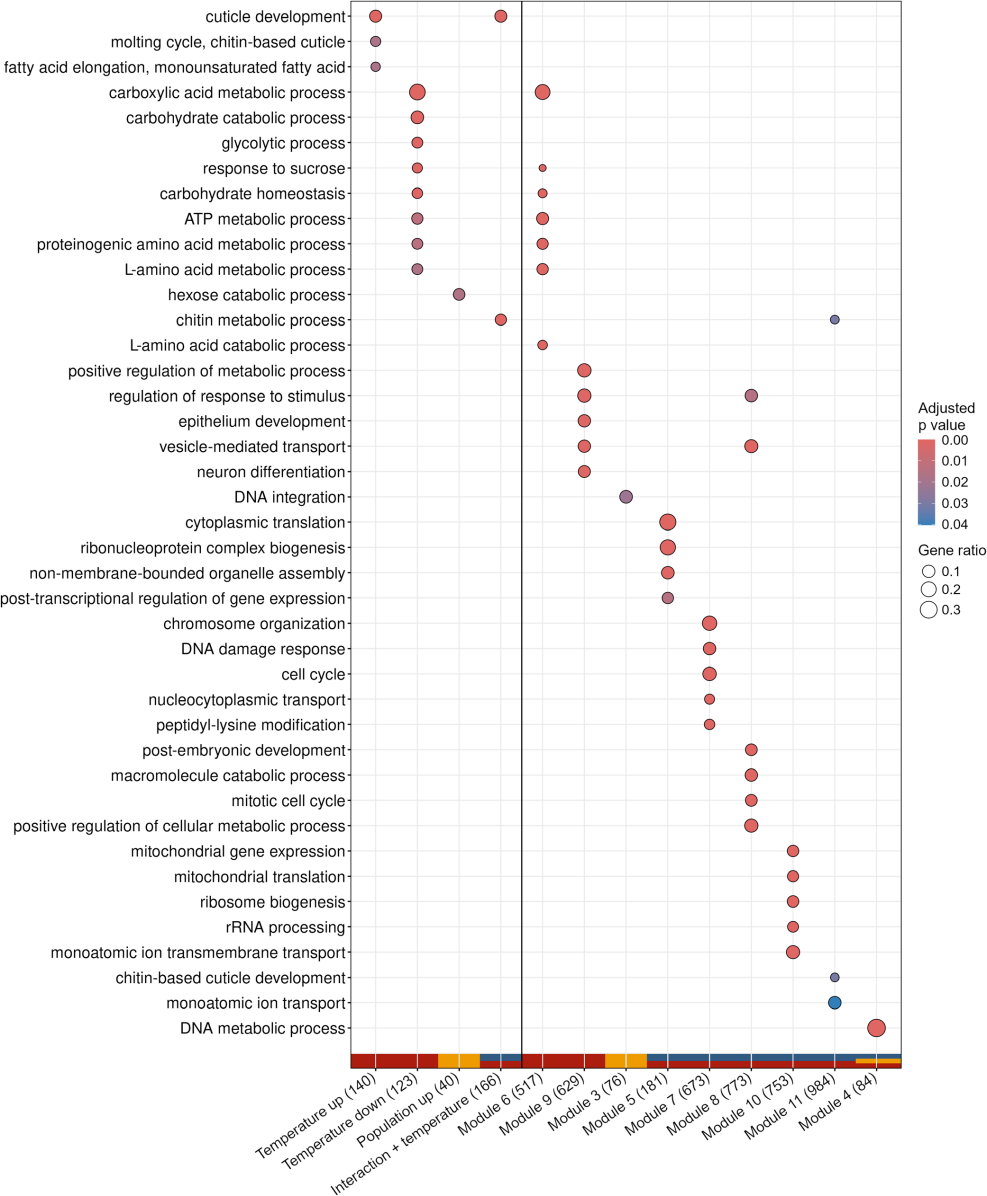
Biological processes overrepresented in differential expression (left of black vertical line) and WGCNA analysis (right of black vertical line). Dot size indicates the ratio between the number of genes within this gene ontology category, and the total number of gene ontology terms found for this differential expression group or co‐expression module. The latter is also indicated in brackets. Dot colour indicates the adjusted *p* value indicating the GO term significance. Colours at the bottom of the plot relate to the Venn diagram in Figure 1 and show significance of temperature (red), population (yellow) and their interaction (blue). The number of non‐overlapping gene ontology terms displayed per significant variable or co‐expression module is restricted to five.

Out of a total of 1686 differentially expressed genes, 469 genes had been assigned to ontology terms for biological processes in the latest Glanville fritillary butterfly genome annotation (Smolander et al. 2022). Similarly, for 9976 genes assigned to co‐expression modules with significant model terms, 3897 genes had corresponding gene ontology terms for biological processes. In general, many pathways related to general development, cellular maintenance and metabolism were differentially expressed and related to co‐expression modules (Figure 3). Overrepresentation analysis on the individual genes differentially expressed with temperature suggests that biological processes related to the exoskeleton (cuticle development, chitin metabolic processes) are upregulated with temperature increase. Contrarily, different processes related to metabolism were downregulated with increasing temperature. From the WGCNA analysis, eigengene expression increased significantly with temperature in module 9 and decreased significantly with temperature in module 6. Biological processes related to module 6 overlap with those downregulated with temperature. For module 9, some extra processes such as metabolic processes, regulation of response to stimulus, vesicle‐mediated transport and neuron differentiation are overrepresented. The only GO terms related to differences between population were hexose catabolic process and DNA integration (module 3). Genes differentially expressed for both the population‐by‐temperature interaction and temperature itself were overrepresented for chitin metabolic processes. Furthermore, modules 5, 7, 8, 10 and 11 were significant for both the interaction and temperature. Processes overrepresented in these modules are related to translation and RNA regulation (modules 5 and 10), cell cycle and metabolism (modules 7 and 8), and the exoskeleton (module 11). One module significant for temperature, population and their interaction was overrepresented for DNA metabolic processes. We did not find any GO terms or enough annotations to describe the functions of genes significant for genetic assimilation or compensation.

## 4 Discussion

The aim of this study was to determine dominant responses in gene expression to increased temperatures in two populations of Glanville fritillary butterflies, from Finland and Spain, using a space‐for‐time approach. We found that most differentially expressed genes (57.5%) and co‐expression modules (8 out of 11) were responsive to temperature, and many of these plastic responses were conserved between populations (32.3% of differentially expressed genes and 2 out of 11 co‐expression modules). However, interactions between temperature and population indicating differences in temperature response between populations were almost equally as prevalent (in 55.1% of genes and 6 out of 11 co‐expression networks), suggesting local adaptation of plastic responses. Surprisingly, the gene‐by‐gene analysis revealed that most temperature‐by‐population interactions showed a reversal in gene expression in response to temperature in Spanish and Finnish populations (30.3% of genes), while most co‐expression modules from the WGCNA analysis with significant temperature‐by‐population interactions did not show such reversals, but only a difference in slope between populations (5 out of 11 co‐expression modules).

### 4.1 Temperature Effects

Firstly, we showed that gene expression was highly plastic in response to temperature for both populations, with 57.5% of all differentially expressed genes and eight out of 11 gene co‐expression modules responding to temperature. Such large effects of temperature have been found across taxa (Gierz et al. 2017; Preston et al. 2022; Seneca and Palumbi 2015; Stanford et al. 2020) and are not unexpected since temperature affects biochemical reaction rates (Brown et al. 2004), which are at the basis of life. These findings show that phenotypic plasticity is present in the Finnish, heat‐sensitive population and retained in the Spanish, heat‐tolerant population and may thus be an important response to future climate warming. While previous studies focusing on the effects of temperature on gene expression have often found upregulation of heat shock proteins (HSPs) with temperature (e.g., Feder and Hofmann 1999; Gehring and Wehner 1995; Günter et al. 2020; Nguyen et al. 2016; Penny and Pavey 2023), HSPs were not overrepresented in any of the gene sets analysed in this study. Furthermore, out of 22 single genes annotated to be related to HSPs, only six were significantly differentially expressed for the population‐by‐temperature interaction. Although better genome annotation will help in shedding more light on this topic, our results may indicate that the temperatures used in our study were not stressful enough to induce HSP activation.

Instead, most overrepresented processes were related to cellular maintenance and growth, metabolism and protein synthesis. Similar results were found in mild warming studies across latitude in heat tolerant seagrass *Nanozostera noltii* (Franssen et al. 2014) and the blue‐tailed damselfly *Ischnura elegans* (Swaegers et al. 2020; Wos et al. 2023). Additionally, genes related to chitin and cuticle development and moulting cycle were upregulated with temperature. These processes are consistent with several studies showing increased developmental rate under warmer conditions in insects (Gillooly et al. 2002; Horne et al. 2015; Verberk et al. 2021), including the Glanville fritillary butterfly (Kvist et al. 2013; Saastamoinen et al. 2013; Verspagen et al. 2023). They might furthermore be related to indirect stressors connected with higher temperatures, such as desiccation stress.

### 4.2 Population by Temperature Effects

GxE interactions were almost equally important as temperature, with 55.1% of differentially expressed genes and six out of 11 gene modules having a significant interaction term. This indicates potential local adaptation in phenotypic plasticity in response to mild warming. In contrast to our results, some previous studies in other insects have found largely parallel reaction norms, where the main effects of temperature or population were responsible for the majority of differentially expressed genes (Clemson et al. 2016; Cooper et al. 2012; Pimsler et al. 2020; Swaegers et al. 2020). Still, significant GxE interactions are common across the literature (Kelly 2019), but patterns on the direction of such differences in plasticity are varied. We found more genes (226 vs. 143) and co‐expression modules (modules 7, 8, 10 and 11 vs. module 5) that are more plastic in the Finnish, heat‐sensitive population compared to the Spanish, heat‐tolerant population. Contrarily, evidence in *Daphnia* shows equal numbers with higher and lower plasticity in the heat‐tolerant population (Yampolsky et al. 2014). In *Drosophila*, heat‐tolerant populations were more plastic than heat‐sensitive ones (Levine et al. 2011).

Two well‐documented GxE responses where plasticity is lost in the heat‐tolerant population are genetic assimilation (Barshis et al. 2013; Franssen et al. 2014; Kenkel et al. 2013) or genetic compensation (Campbell‐Staton et al. 2021; Koch and Guillaume 2020; Monroe et al. 2016; Swaegers et al. 2020). In the case of genetic assimilation, the initial plastic response is beneficial and becomes fixed in the heat‐tolerant population that is more regularly exposed to warm conditions, while the heat‐sensitive population retains the plastic trait (Loison 2019; Pigliucci et al. 2006). On the other hand, genetic compensation occurs when plasticity is maladaptive, resulting in less plasticity in the heat‐tolerant population with expression levels across the entire temperature range similar to those before warming in the evolved population (Grether 2005). Despite the loss of plasticity in the Finnish population, we found signs for genetic assimilation or compensation only in 1.5% and 2.4% of differentially expressed genes respectively and in none of the co‐expression modules. Not enough annotation terms were present to interpret functions of these genes. Theory has predicted that genetic assimilation might be unlikely to occur because the time needed for such evolution might exceed the time the environment might be stable (Scheiner et al. 2017), but more research is thus needed to better understand which circumstances will cause assimilation or compensation. It is worth noting that while this cannot be detected, the flatter reaction norms in the Spanish population in the gene‐by‐gene analysis may point to partial assimilation or compensation. Similarly, reaction norms of co‐expression modules 7 and 11 are less steep in the Spanish population, and gene expression in this population is similar to that of 25°C across the range, indicating partial compensation. Although our data does not provide certain evidence on exactly how often partial compensation or assimilation might occur, the lower levels of phenotypic plasticity in the Spanish, heat‐tolerant population indicate that some forms of plasticity might be detrimental in scenarios of climate warming. This would leave especially northern populations not yet adapted to warm climates vulnerable to future climate change and should be further investigated.

In the gene‐by‐gene differential expression analysis, we additionally found a reversal of reaction norms, where significant GxE interactions without main effects of temperature or population are present in 30.3% of differentially expressed genes. To our knowledge, such reversals are uncommon in the literature. Reaction norm reversals could indicate trade‐offs or imply that populations have divergent strategies for coping with increased temperature. In our previous study (Verspagen et al. 2023), we found a difference in life‐history strategy between Spanish and Finnish populations in response to colder rearing conditions, which might reflect adaptations to differences in growing season length. While the Spanish population had a long development time but grew large in the colder rearing temperature, Finnish individuals had a short development time but were smaller. This difference in life‐history strategy might explain the reversal in gene expression in response to temperature between the two populations found in a set of genes in the current study. However, since it was not possible to measure phenotypic traits directly in the same individuals used for gene expression studies, such connections need to be interpreted with caution. Future research could include performing phenotypic measurements and RNA extractions from the same individuals to better draw connections between gene expression and phenotype. Notably, in Verspagen et al. (2023), responses diverged especially at the lower temperature, potentially indicating adaptation to cold rather than heat. We cannot be certain whether the current results are caused by adaptation to heat or cold or other factors differing across the latitudinal cline such as precipitation, biotic interactions, or demographic histories. However, gene expression is especially divergent at high temperature, suggesting heat might likely be the driving factor for adaptation. Somewhat surprisingly, we did not find similar reaction norm reversals in the WGCNA analysis. This suggests that the reversal is clear in a small set of genes with potential large effect but does not reflect coordinated low‐level responses. Further research into the functions of reversal genes could shed more light on this.

Eigengene expression in three out of six co‐expression modules with significant population by temperature interaction terms (modules 8, 10 and 11) was bimodally distributed at the highest temperature treatment. Gene expression in these modules thus responds to temperature in a discrete matter, with response in different individuals fitting in one of two modes. These modes are similar between populations, but the GxE interaction indicates that frequencies in each mode differ between populations. This potentially suggests that the thermal reaction norm is not linear but responds to a critical threshold, upon which a regulatory switch is turned. Intraspecific variation in the threshold temperature might explain population differences in reaction norms. Threshold patterns occur often in traits such as morphological states (e.g., seasonally different colour patterns, wing vs. wingless forms), diapause induction or other life history decisions, sex, etc. (e.g., Bégin and Roff 2002; Debes et al. 2020; Hazel et al. 2004; Reid and Acker 2022; Roff 1994). However, the threshold pattern in our data does not follow the same pattern as phenotypic developmental data collected in Verspagen et al. (2023), where population divergence took place especially at the coldest test temperature. This could indicate that diverging gene expression patterns are needed in the two populations to reach similar phenotypic outcomes. Future research should add intermediate test temperatures to further investigate this pattern. Due to the nature of gene‐by‐gene differential expression analysis, where expression of large numbers of genes are tested separately, patterns in distribution of gene expression as found here are hard to detect with this method. This highlights the added value of combining different analysis methods to obtain a fuller picture of gene expression patterns.

### 4.3 Sex Effects

Sex is often a strong factor underlying differential gene expression (Ellegren and Parsch 2007), yet we found that only six genes were significantly differentially expressed between the two sexes. While two co‐expression modules showed significant differences between sexes, effect sizes were small. Sex therefore does not seem to be an important factor in our data. Sex‐specific expression of genes is only expected in certain tissues (Grath and Parsch 2016; Parisi et al. 2003), therefore, by sampling whole bodies we may have missed important sex‐specific responses to temperature. However, sex‐specific gene expression is also known to differ across developmental stages (Grath and Parsch 2016; Perry et al. 2014), and because larvae were sampled at an early stage in development, sex‐specific expression differences may not have been present.

## 5 Conclusions

Overall, we found numerous genes that are differentially expressed during larval development in response to temperature, while differences in mean trait values between populations were less common. This indicates that phenotypic plasticity is largely conserved as a response to mild warming. Still, expression of many genes was also affected by a significant GxE interaction. Although the Finnish, heat‐sensitive population generally was more responsive to temperature than the Spanish, heat‐tolerant population, we did not find many genes exhibiting genetic assimilation or compensation. Together, these results suggest that both plasticity and evolution are an important response to warming, but selection acts on slopes of reaction norms rather than mean trait values. We found considerable differences in the number of genes affected by our explanatory variables between gene‐by‐gene differential expression (1715 genes) and WGCNA analysis (10,156 genes). This confirms that while differential expression analysis identifies single genes with large expression differences, WGCNA distinguishes smaller but coordinated differences (Orsini et al. 2018; Stanford and Rogers 2018). Furthermore, both analyses revealed gene expression patterns not detected by the other. This highlights that combining different analysis techniques provides a more complete picture of gene expression patterns. Biological processes related to differentially expressed genes with temperature as well as GxE are mostly related to regular cellular maintenance and growth, as was expected from phenotypic studies. However, the function of many differentially expressed genes found in our study remains unknown, and we thus call for further research investigating these unknown genes. Furthermore, future research should focus on more populations and temperatures, and include measures of fitness. This would increase understanding of patterns of phenotypic plasticity and adaptation and how they interact across different environments. Our results highlight that adequate genetic variation underlying plastic traits might be important for species to continue to adapt to climate change.

## Author Contributions

N.V., M.S. and M.F.D. conceptualised the project and designed the methodology. M.F. extracted RNA for sequencing and DNA for the sex determination. N.V., R.D.R. and H.M. analysed the data, and N.V. did the data visualisation. N.V. wrote the original draft with contribution from M.F., R.D.R., H.M. and M.F.D. All co‐authors contributed to reviewing and editing the final manuscript.

## Funding

This work was supported by the Natural Sciences and Engineering Research Council of Canada, Fellowship; Research Council of Finland, 316227; LUOVA doctoral programme: Salaried position.

## Disclosure

Benefits generated: Benefits from this research accrue from the sharing of our data and results on public databases as described above.

## Ethics Statement

All the research complies with applicable laws on sampling from natural populations and animal experimentation (including the ARRIVE guidelines).

## Conflicts of Interest

The authors declare no conflicts of interest.

## Supporting information


**Appendix S1:** mec70309‐sup‐0001‐AppendixS1.docx.

## Data Availability

The raw sequencing data for this study have been deposited in the European Nucleotide Archive (ENA) at EMBL‐EBI under accession number PRJEB110919. Count and sample metadata and scripts used for analysis can be found from Dryad at the following DOI: https://doi.org/10.5061/dryad.4xgxd25qj. Reference genome and annotations were downloaded from Smolander et al. (2022): https://doi.org/10.5524/100915. The nf/core script used for alignment of sequencing data to the reference genome can be downloaded from here: https://nf‐co.re/rnaseq/3.11.2.
