# Supplementary material for: Mild Warming Induces Divergent Plastic Responses in Gene Expression Among Populations of a Temperate Butterfly
Source: Mol Ecol. 2026 Apr 1;35(7):e70309. doi: 10.1111/mec.70309 (PMC13044392; doi:10.1111/mec.70309)
Supplement: Supplementary file 1 — Appendix S1: mec70309‐sup‐0001‐AppendixS1.docx. [file MEC-35-e70309-s001.docx]

SUPPLEMENTARY MATERIALS FOR

Mild warming induces divergent plastic responses in gene expression among populations of a temperate butterfly.

CONTENTS

Figure S1: Experimental family selection

Figure S2: Different methods for variance stabilisation

Figure S3: Dendrogram showing the sample clustering

Figure S4: PCA exploratory plot

Figure S5: Selection of soft-thresholding power in WGCNA

Figure S6: WGCNA module dendrogram

Figure S7: Bimodal indices for WGCNA eigengenes

Figure S8: WGCNA Eigengene expression for different sexes

Table S1: Sampling locations

Table S2: Information on primers used for sex determination

Table S3: WGCNA eigengene expression quantile regression results


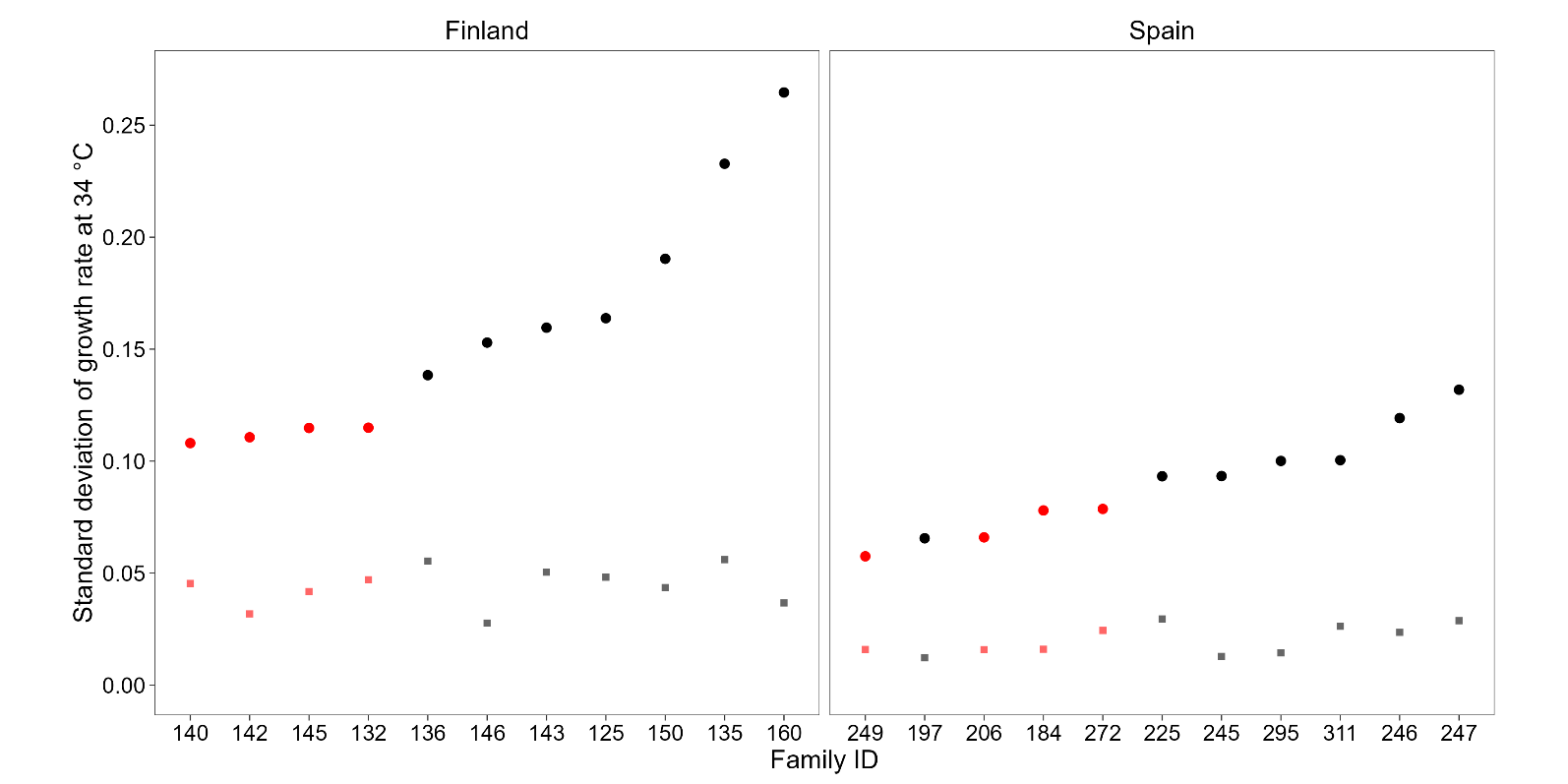


**Figure S1**. Selection of families for RNA sequencing. Circles indicate the standard deviation of growth rate at 34 °C. Squares show slope of the growth rate reaction norm. Points in red were chosen for this experiment.

**
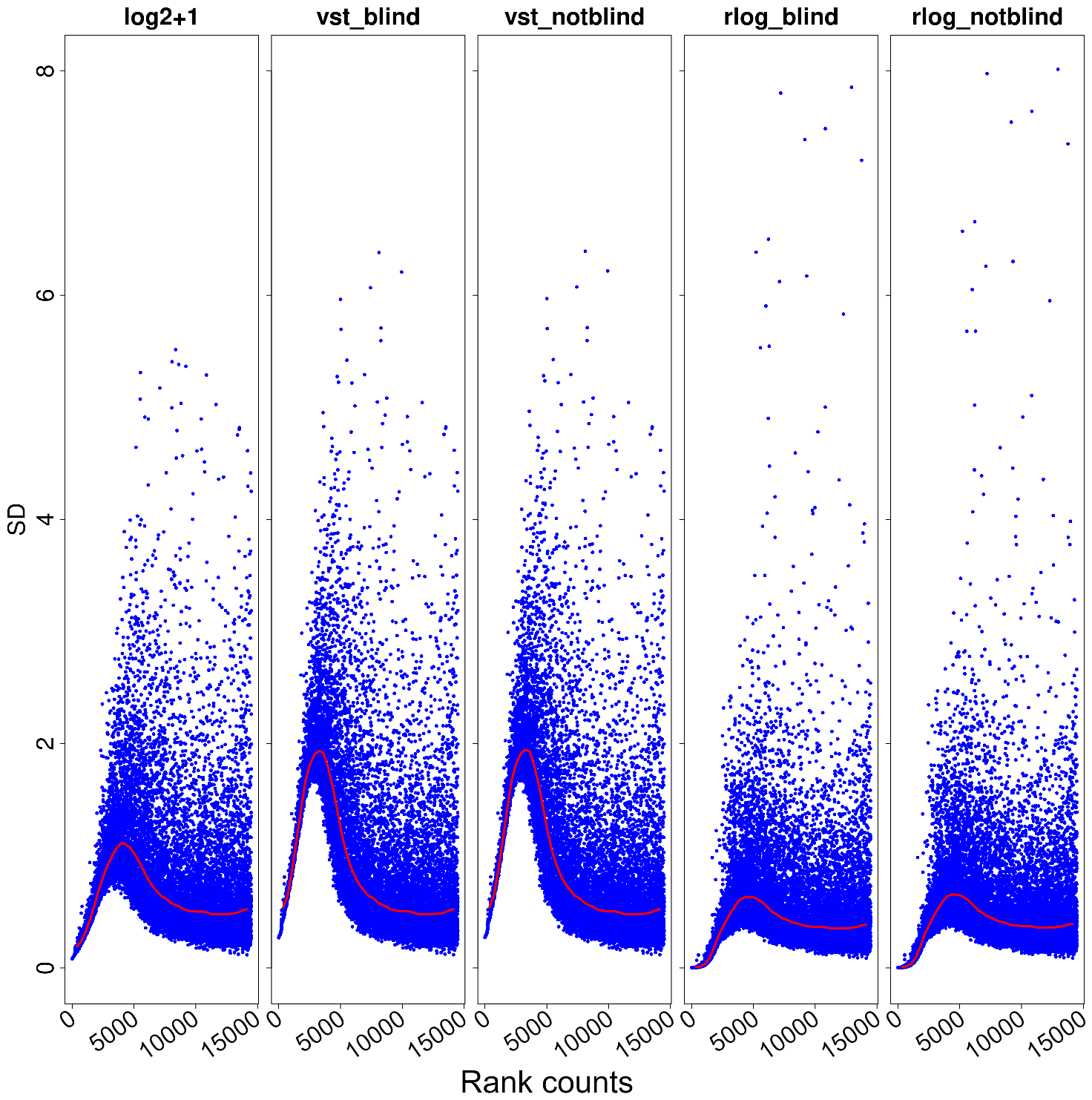
**

**Figure S2**. The results of the variance stabilizing normalization step before WGCNA. The x-axis shows the rank of gene counts and the y-axis standard deviation of gene counts. Five different normalization methods were tested: log2 of the raw counts plus pseudo count, variance stabilizing normalization without (vst_blind) or with (vst_notblind) taking into account temperature and population effects, and rlog normalization without (rlog_blind) or with (rlog_notblind) taking into account temperature and population effects. All normalization steps were calculated as implemented in the R package DeSeq2.


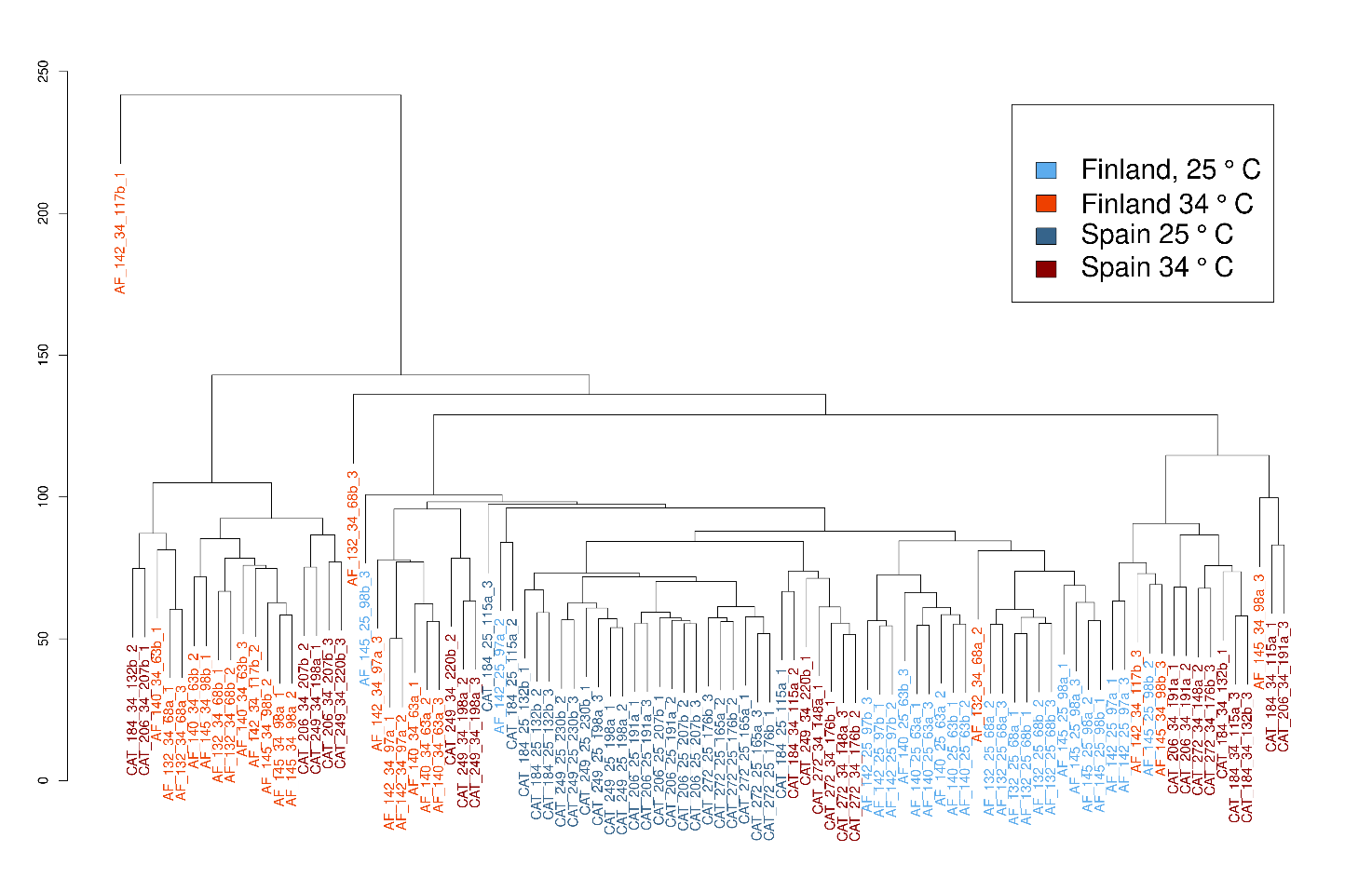


**Figure S3**. Dendrogram showing the sample clustering based on all genes after removing 313 genes with zero variance and rlog normalization. Based on this dendrogram, one sample from Finland in the 34°C treatment was considered an outlier and removed from further analysis.


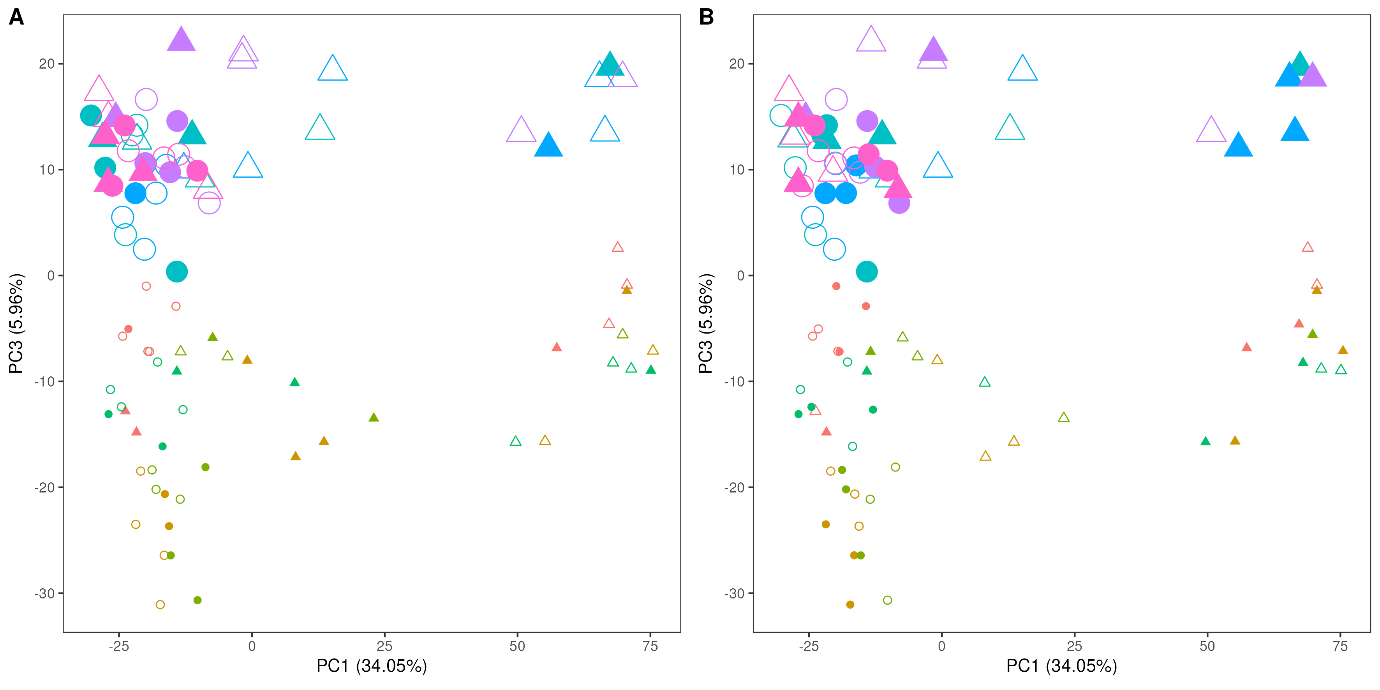


**Figure S4**. PCA plot with 2000 most differentially expressed genes showing PC1 and PC3, that explain 34.05% and 5.96% of variance respectively. Point size indicates population (large points for Spain and small points for Finland). Point shape represents the temperature treatment (circles for 25°C, triangles for 34°C). Different families are represented by different colours. In (**A**), open symbols show females, and closed symbols show males. In (**B**), symbol fill types (open or closed) represent the two replicate rearing groups.


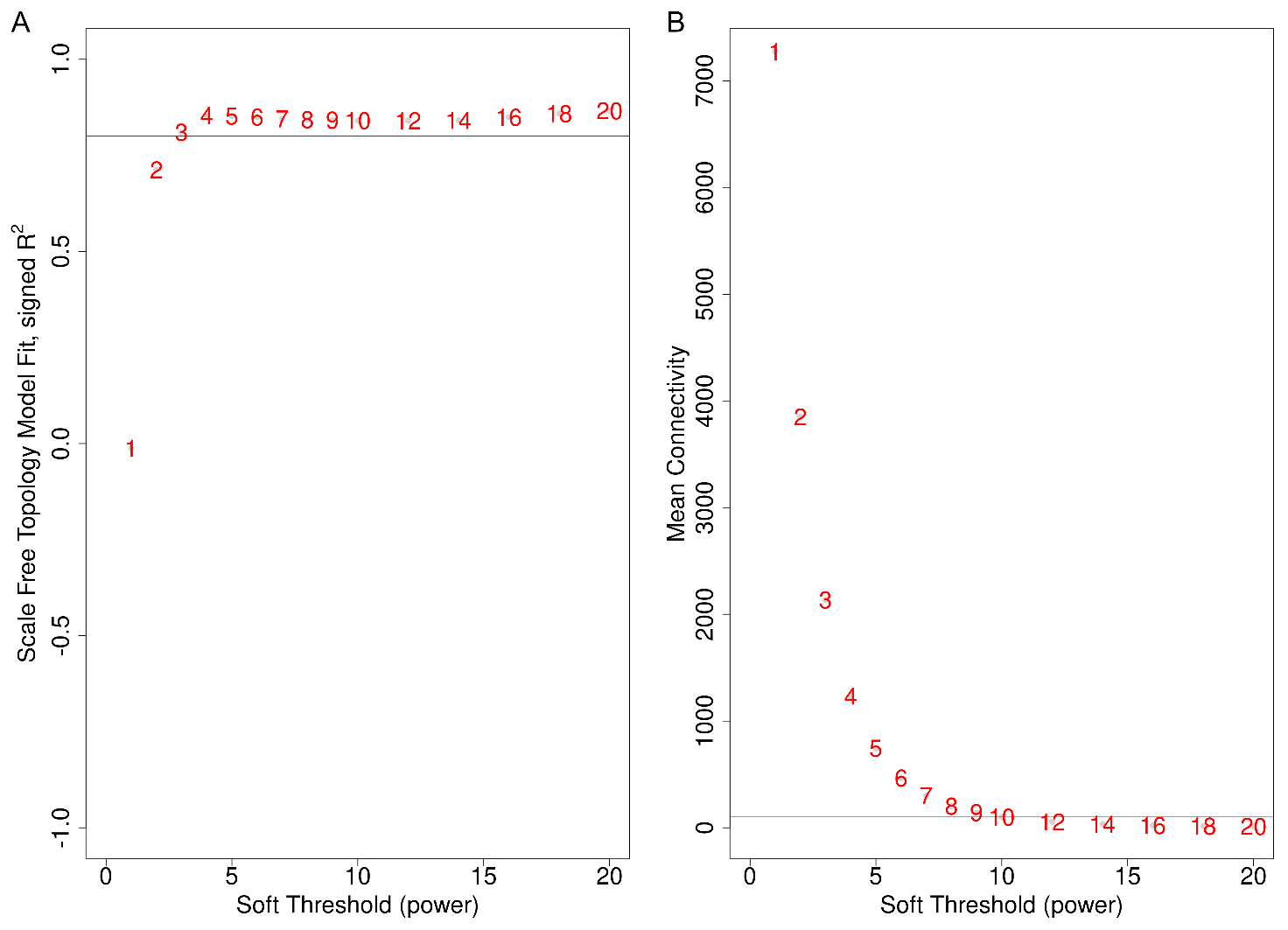


**Figure S5**. The selection of soft-thresholding power in WGCNA. The scale independence (**A**) shows the soft threshold power as a function of signed R^2^ correlation. The green line corresponds to R^2^ = 0.8, and the picked threshold should be above this value, as well as start to plateau. **B** shows the mean connectivity, i.e. the sum of correlations across all samples as a function of the soft threshold, and the soft threshold should be above 100 (green line). As a result, a soft threshold power of 4 was used in the construction of the weighted gene co-expression analysis.


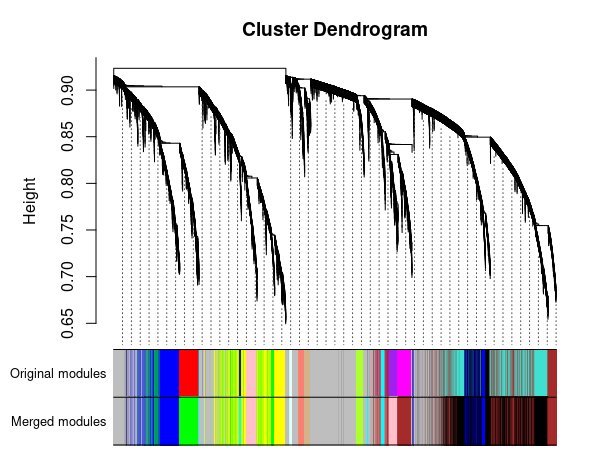


**Figure S6**. The result of the WGCNA analysis shown as a dendrogram and the corresponding modules (here labeled as colors) using the dynamic tree cut method. Top row (Dynamic Tree Cut) shows the modules before merging, while the bottom row (Merged Dynamic) shows merged modules.


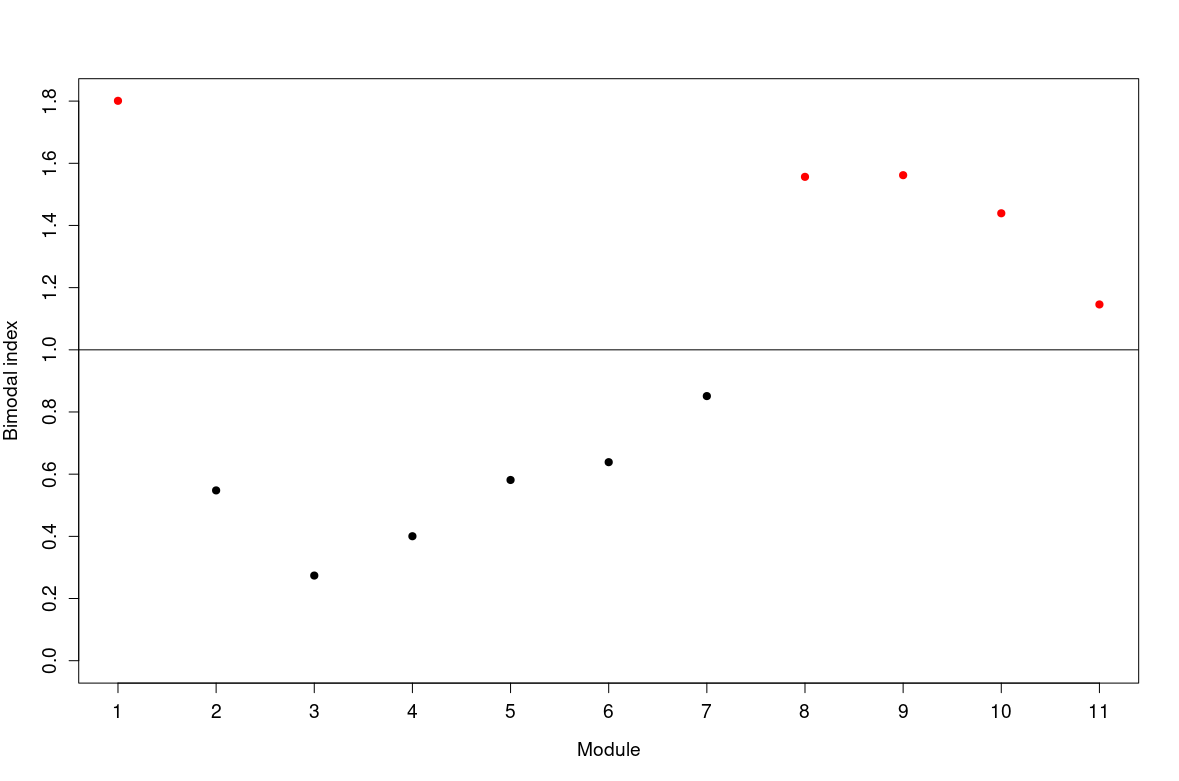


**Figure S7**. Bimodal indices for 11 gene expression modules. All modules with a bimodal index above one (black line) were treated as bimodal in subsequent quantile regression analyses.

**
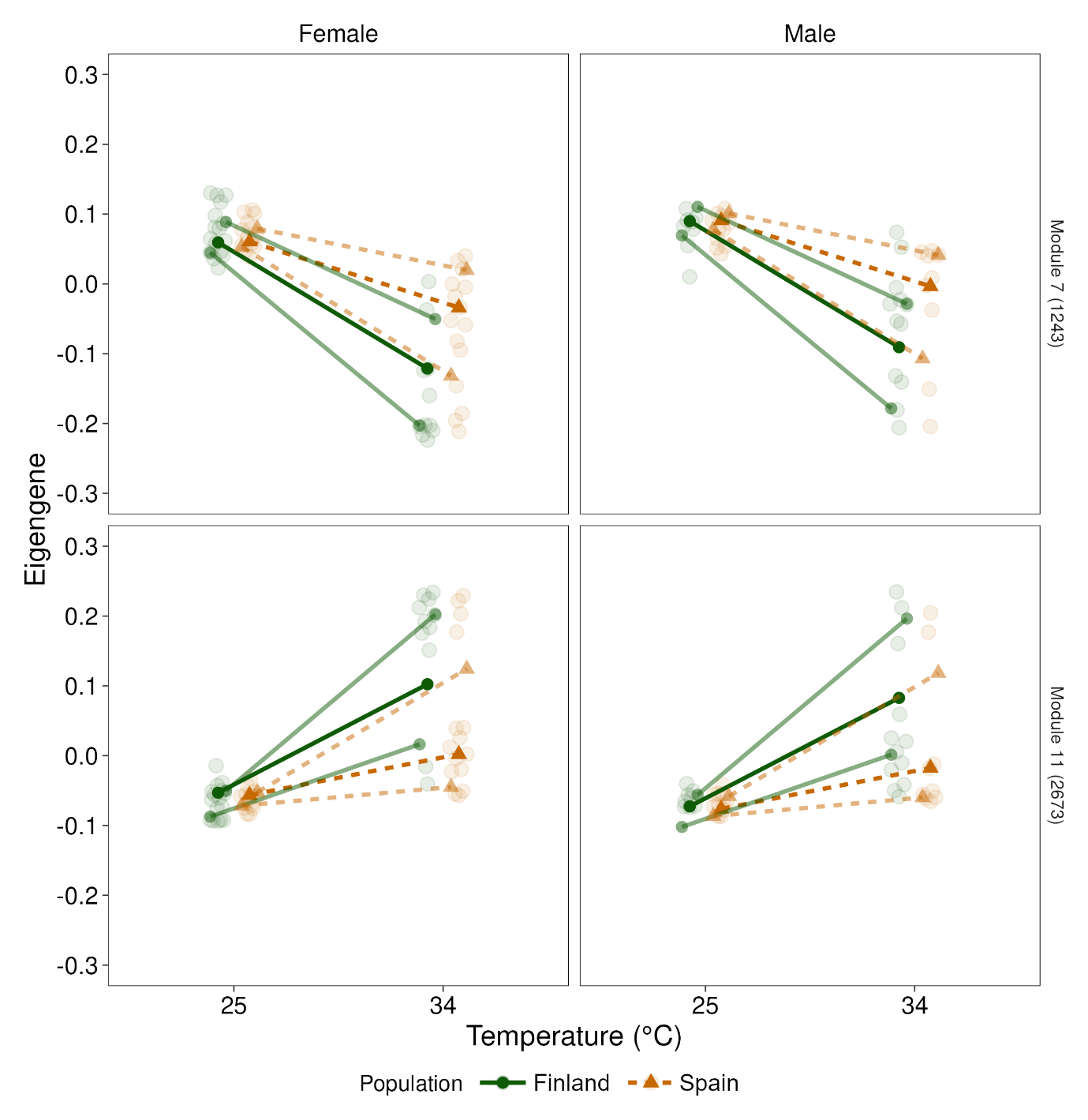
**

**Figure S8**. Eigengene expression for each temperature, population and sex for the two modules (Module 7 and 11) that were significantly differentially expressed for sex. Dark green circles and solid lines represent the Finnish population, brown triangles and dashed lines represent the Spanish population. Transparent points in the background indicate raw eigengene expression and solid points in the foreground connected by lines show mean predicted values for the 0.5 quantile. Semi-transparent points and lines show mean predicted values for the 0.25 and 0.75 quantiles.

**Table S1**: Detailed location of collection sites and periods of F0 generation for each area along the latitudinal cline.

| **Collection period** | **♀ ID** | **Longitude mother** | **Latitude mother** | **Longitude father** | **Latitude father** |
| --- | --- | --- | --- | --- | --- |
| **Finland** | | | | | |
| September 2019 | 140 | 20.13002 | 60.28681 | 19.80119 | 60.3346 |
|  | 132 | 19.59276 | 60.22811 | 19.92981 | 60.37673 |
|  | 142 | 19.96278 | 60.34853 | 20.1249 | 60.22045 |
|  | 145 | 20.08041 | 60.11084 | 20.18572 | 60.21992 |
| **Spain** | | | | | |
| May 2019 | 184 | 2.02020 | 42.02220 | 1.78000 | 42.11209 |
|  | 272 | 2.72963 | 41.79945 | 2.02020 | 42.02220 |
|  | 206 | 1.85024 | 42.11491 | 1.78000 | 42.11209 |
|  | 249 | 2.29413 | 41.81226 | 2.72963 | 41.79945 |

**Table S2**. Information on primers used for sex determination

| Primer ID | Assembly | Chrom | SNP POS | REF | ALT | Heterozygous | Reads depth | F_overhangs | Forward sequences 5’ -> 3’ | R_overhangs | Reverse sequences 5’ -> 3’ | Amplicon size (bp) | Pool | Reference |
| --- | --- | --- | --- | --- | --- | --- | --- | --- | --- | --- | --- | --- | --- | --- |
| koe532 | ilMelCinx1.1 (GCF_905220565.1) | NC_059424.1 | 1931691 | A | G | Åland/Spain |  | ACACTCTTTCCCTACACGACGCTCTTCCGATCT | TGGTAAACTGTCTTACCAGC | GTGACTGGAGTTCAGACGTGTGCTCTTCCGATCT | TTCTCCCTCAACAGTTGTCC | 84 | B | Kahilainen et al., 2022 |
| s523_125810 | ilMelCinx1.1 (GCF_905220565.1) | NC_059424.1 | 2545684 | C | T | Åland/Spain |  | ACACTCTTTCCCTACACGACGCTCTTCCGATCT | AGTAGGCAGAGAAGAACCAG | GTGACTGGAGTTCAGACGTGTGCTCTTCCGATCT | GCATTTCAGAGTGCTTAGCC | 77 | A | Kahilainen et al., 2022 |
| POS_2824213 | ilMelCinx1.1 (GCF_905220565.1) | NC_059424.2 | 3124753 | T | C | Spain |  | ACACTCTTTCCCTACACGACGCTCTTCCGATCT | GGTCCGTGCAGTTTCTCTTC | GTGACTGGAGTTCAGACGTGTGCTCTTCCGATCT | CCGTGACCATTCCGTATTCA | 86 | A | unpublished data |
| s2188_256349 | ilMelCinx1.1 (GCF_905220565.1) | NC_059424.3 | 3831528 | G | T | Åland | low (Spain) | ACACTCTTTCCCTACACGACGCTCTTCCGATCT | AATGAATGTGCGATTCCATC | GTGACTGGAGTTCAGACGTGTGCTCTTCCGATCT | ACCCACACTTTCCTGTTTCG | 95 | A | Kahilainen et al., 2022 |
| POS_6409349 | ilMelCinx1.1 (GCF_905220565.1) | NC_059424.4 | 6785940 | C | T | Åland/Spain |  | ACACTCTTTCCCTACACGACGCTCTTCCGATCT | TAATGAGCTACAATAAGAGCGG | GTGACTGGAGTTCAGACGTGTGCTCTTCCGATCT | TGCTTCTCCAGTGAGTTGAC | 108 | A | unpublished data |
| koe303 | ilMelCinx1.1 (GCF_905220565.1) | NC_059424.1 | 6837586 | G | A | Åland |  | ACACTCTTTCCCTACACGACGCTCTTCCGATCT | ACAGGTACAGGATGTGTGTC | GTGACTGGAGTTCAGACGTGTGCTCTTCCGATCT | ACAATCGGCAAGTGCTCAAG | 86 | B | Kahilainen et al., 2022 |
| POS_8957501 | ilMelCinx1.1 (GCF_905220565.1) | NC_059424.1 | 9271372 | G | A | Åland/Spain |  | ACACTCTTTCCCTACACGACGCTCTTCCGATCT | CGTTCGCTATTGTCGGTTGAG | GTGACTGGAGTTCAGACGTGTGCTCTTCCGATCT | GTTTCTCCGTGAATACGAGCC | 127 | A | unpublished data |
| s4145_101448 | ilMelCinx1.1 (GCF_905220565.1) | NC_059424.1 | 9299611 | C | T | Åland |  | ACACTCTTTCCCTACACGACGCTCTTCCGATCT | TCGATGATATCAGAAGCCGC | GTGACTGGAGTTCAGACGTGTGCTCTTCCGATCT | AGTACGCATCTATCGCAGAC | 95 | B | Kahilainen et al., 2022 |
| FLN63_SNPK1 | ilMelCinx1.1 (GCF_905220565.1) | NC_059424.1 | 9872250 | C | A | Åland |  | ACACTCTTTCCCTACACGACGCTCTTCCGATCT | GATGTGGTAGAAGAAGACGC | GTGACTGGAGTTCAGACGTGTGCTCTTCCGATCT | AGTTTGTTCTGGAGCTGCAC | 90 | B | Kahilainen et al., 2022 |
| POS_10298113 | ilMelCinx1.1 (GCF_905220565.1) | NC_059424.1 | 10549851 | C | T | Spain |  | ACACTCTTTCCCTACACGACGCTCTTCCGATCT | ACTTTCAGCTACGGAACGTG | GTGACTGGAGTTCAGACGTGTGCTCTTCCGATCT | CTCGGAAATCTCTAATCGACGTG | 119 | A | unpublished data |
| s1819_91872 | ilMelCinx1.1 (GCF_905220565.1) | NC_059424.1 | 10593707 | T | C | Åland |  | ACACTCTTTCCCTACACGACGCTCTTCCGATCT | CTGAAAAGGTAGCATCAGGC | GTGACTGGAGTTCAGACGTGTGCTCTTCCGATCT | ACCATGGTAACCACTCGATG | 87 | B | Kahilainen et al., 2022 |
| POS_11418474 | ilMelCinx1.1 (GCF_905220565.1) | NC_059424.1 | 11633349 | T | C | Spain |  | ACACTCTTTCCCTACACGACGCTCTTCCGATCT | CGACAACCATGAAGGCACTG | GTGACTGGAGTTCAGACGTGTGCTCTTCCGATCT | TTGTTCGGAGCTAGTGATTAGG | 77 | B | unpublished data |
| s3244_500584 | ilMelCinx1.1 (GCF_905220565.1) | NC_059424.1 | 13322674 | T | C | Åland/Spain |  | ACACTCTTTCCCTACACGACGCTCTTCCGATCT | GGTACTCTTTTAAGTTTGGCG | GTGACTGGAGTTCAGACGTGTGCTCTTCCGATCT | CGGTATGTCGTACTATTTCG | 84 | B | Kahilainen et al., 2022 |
| POS_17051852 | ilMelCinx1.1 (GCF_905220565.1) | NC_059424.1 | 17232151 | T | C | Åland/Spain |  | ACACTCTTTCCCTACACGACGCTCTTCCGATCT | CTAGGTGTTCCTTAATGCCG | GTGACTGGAGTTCAGACGTGTGCTCTTCCGATCT | TATTGGAGGTGGAGTGGCAG | 134 | B | unpublished data |
| s2428_53537 | ilMelCinx1.1 (GCF_905220565.1) | NC_059424.1 | 17269396 | G | A | Åland |  | ACACTCTTTCCCTACACGACGCTCTTCCGATCT | GGTCGAATGTACGGTCGTTT | GTGACTGGAGTTCAGACGTGTGCTCTTCCGATCT | GCCGAAACAACTAAAGCCAC | 77 | A | Kahilainen et al., 2022 |
| Lap_461Y | ilMelCinx1.1 (GCF_905220565.1) | NC_059424.1 | 22165862 | A | T | Åland |  | ACACTCTTTCCCTACACGACGCTCTTCCGATCT | TTTGAGATTGCGCCTTTAAGGT | GTGACTGGAGTTCAGACGTGTGCTCTTCCGATCT | CCAGTAAGGAAATTGTTCATCCAG | 96 | A | unpublished data |
| Lap_461Y | ilMelCinx1.1 (GCF_905220565.1) | NC_059424.1 | 22165874 | G | A | Åland/Spain |  | ACACTCTTTCCCTACACGACGCTCTTCCGATCT | TTTGAGATTGCGCCTTTAAGGT | GTGACTGGAGTTCAGACGTGTGCTCTTCCGATCT | CCAGTAAGGAAATTGTTCATCCAG | 96 | A | unpublished data |
| Lap_207Y | ilMelCinx1.1 (GCF_905220565.1) | NC_059424.1 | 22166063 | A | G | Åland |  | ACACTCTTTCCCTACACGACGCTCTTCCGATCT | AAACTTTTGAAGCGCCTGTG | GTGACTGGAGTTCAGACGTGTGCTCTTCCGATCT | CAACCTTGAACAATTAGACG | 79 | B | Kahilainen et al., 2022 |
| Pro_490S | ilMelCinx1.1 (GCF_905220565.1) | NC_059424.1 | 22170737 | C | T | Åland |  | ACACTCTTTCCCTACACGACGCTCTTCCGATCT | TTCAGCCAGACATTCCAGCC | GTGACTGGAGTTCAGACGTGTGCTCTTCCGATCT | TGACATTGGACTGGGAACCG | 96 | B | unpublished data |
| Pro_192Y_v2 | ilMelCinx1.1 (GCF_905220565.1) | NC_059424.1 | 22170977 | A | G | Åland/Spain |  | ACACTCTTTCCCTACACGACGCTCTTCCGATCT | TCGTCGACGCTTATCGGTTG | GTGACTGGAGTTCAGACGTGTGCTCTTCCGATCT | CCTAAAAACAACGGTCGTGG | 75 | B | Kahilainen et al., 2022 |
| Kettin_K117 | ilMelCinx1.1 (GCF_905220565.1) | NC_059424.1 | 22288938 | G | T | Åland |  | ACACTCTTTCCCTACACGACGCTCTTCCGATCT | CACTGTGTCTTCCACCTCTC | GTGACTGGAGTTCAGACGTGTGCTCTTCCGATCT | ACTTTGTTCATTGCTGCTTCC | 113 | A | unpublished data |
| Kettin_Y255 | ilMelCinx1.1 (GCF_905220565.1) | NC_059424.1 | 22289079 | A | C | Åland/Spain |  | ACACTCTTTCCCTACACGACGCTCTTCCGATCT | AGCTTGTCGAAGGTCAACAC | GTGACTGGAGTTCAGACGTGTGCTCTTCCGATCT | CCAGTAGTCAAAGAAACTCCGT | 105 | A | unpublished data |
| Titin1_R609 | ilMelCinx1.1 (GCF_905220565.1) | NC_059424.1 | 22325004 | G | A | Åland |  | ACACTCTTTCCCTACACGACGCTCTTCCGATCT | TGTAGCTGATGGCAAAGTAG | GTGACTGGAGTTCAGACGTGTGCTCTTCCGATCT | GATGATGTACAAAAGGTGCTG | 80 | B | Kahilainen et al., 2022 |
| Titin2_408Y | ilMelCinx1.1 (GCF_905220565.1) | NC_059424.1 | 22365797 | C | T | Åland |  | ACACTCTTTCCCTACACGACGCTCTTCCGATCT | AGGAAACGAAGACGAAGTTG | GTGACTGGAGTTCAGACGTGTGCTCTTCCGATCT | TCCTTGACCTCGAAGTCTTG | 79 | A | Kahilainen et al., 2022 |
| s734_292342_v2 | ilMelCinx1.1 (GCF_905220565.1) | NC_059424.1 | 15720131 | G | A | NA | low | ACACTCTTTCCCTACACGACGCTCTTCCGATCT | GTAGAAAAGTTCGATAGAGGG | GTGACTGGAGTTCAGACGTGTGCTCTTCCGATCT | GACGCCTTGATATCAATTTG | 76 | A | Kahilainen et al., 2022 |
| s1764_157059 | ilMelCinx1.1 (GCF_905220565.1) | NC_059424.1 | 19526954 | no SNP | no SNP | NA | low | ACACTCTTTCCCTACACGACGCTCTTCCGATCT | CATCTCATCTACATCTCTCC | GTGACTGGAGTTCAGACGTGTGCTCTTCCGATCT | CAATGCGGAAAAACGAGTGG | 104 | A | Kahilainen et al., 2022 |
| TRII_F37_SNPR1 | ilMelCinx1.1 (GCF_905220565.1) | NC_059424.1 | 17072092 | G | A | NA | low | ACACTCTTTCCCTACACGACGCTCTTCCGATCT | CTAAGCAAATGTTGAAGCAG | GTGACTGGAGTTCAGACGTGTGCTCTTCCGATCT | ACGATTACAATACGACACGC | 129 | A | Kahilainen et al., 2022 |
| s2873_41671 | ilMelCinx1.1 (GCF_905220565.1) | NC_059424.1 | 4126819 | C | A | NA | low | ACACTCTTTCCCTACACGACGCTCTTCCGATCT | CCGTAACTCCCATGCAAGAA | GTGACTGGAGTTCAGACGTGTGCTCTTCCGATCT | GTTTTCACGAGATATGGTGC | 126 | A | Kahilainen et al., 2022 |
| Kettin_Y478 | ilMelCinx1.1 (GCF_905220565.1) | NC_059424.1 | 22289277 | C | T | NA | low | ACACTCTTTCCCTACACGACGCTCTTCCGATCT | GCAACAAATTTAATGGGCGA | GTGACTGGAGTTCAGACGTGTGCTCTTCCGATCT | GATACGGTCTAAAGATTCGGG | 104 | A | unpublished data |
| Pro_309R | ilMelCinx1.1 (GCF_905220565.1) | NC_059424.1 | 22170869 | C | T | NA |  | ACACTCTTTCCCTACACGACGCTCTTCCGATCT | TCACCGATGTTATTCTTTGCGT | GTGACTGGAGTTCAGACGTGTGCTCTTCCGATCT | CAGTGTTAAAGATTACTTCGGCTC | 75 | A | unpublished data |
| s201_52818 | ilMelCinx1.1 (GCF_905220565.1) | NC_059424.1 | 13288436 | G | A | NA | low | ACACTCTTTCCCTACACGACGCTCTTCCGATCT | CACTGGATACTCGGATTATG | GTGACTGGAGTTCAGACGTGTGCTCTTCCGATCT | GTCACGGTAAAATCCTAGTC | 115 | B | Kahilainen et al., 2022 |
| s4085_23128 | ilMelCinx1.1 (GCF_905220565.1) | NC_059424.1 | 10105762 | T | C | NA | low | ACACTCTTTCCCTACACGACGCTCTTCCGATCT | TTAACTACAACCACCGCGCC | GTGACTGGAGTTCAGACGTGTGCTCTTCCGATCT | GCTTAAACTAGGCGGTACTG | 117 | B | Kahilainen et al., 2022 |
| s31_204583 | ilMelCinx1.1 (GCF_905220565.1) | NC_059424.1 | 11806247 | T | C | NA | low | ACACTCTTTCCCTACACGACGCTCTTCCGATCT | TGTGCCTTTAGACAACTCAG | GTGACTGGAGTTCAGACGTGTGCTCTTCCGATCT | AACCTCTAATTCAGCATAC | 118 | B | Kahilainen et al., 2022 |
| s2308_193256_v2 | ilMelCinx1.1 (GCF_905220565.1) | NC_059424.1 | 1212135 | no SNP | no SNP | NA | low | ACACTCTTTCCCTACACGACGCTCTTCCGATCT | CTGAACGACGCCAAAAATAC | GTGACTGGAGTTCAGACGTGTGCTCTTCCGATCT | TGTACGTTTGTCTCGAATA | 121 | B | Kahilainen et al., 2022 |
| POS_1453649 | ilMelCinx1.1 (GCF_905220565.1) | NC_059424.1 | 1711064 | C | T | NA | low | ACACTCTTTCCCTACACGACGCTCTTCCGATCT | GAACACGTACGAGCTGGC | GTGACTGGAGTTCAGACGTGTGCTCTTCCGATCT | CGTAACCGCGTCTATTTGGG | 108 | A | unpublished data |
| POS_5729509 | ilMelCinx1.1 (GCF_905220565.1) | NC_059424.1 | 6124887 | A | T | NA | low | ACACTCTTTCCCTACACGACGCTCTTCCGATCT | AGAGCAAATACGTCTTTCGTG | GTGACTGGAGTTCAGACGTGTGCTCTTCCGATCT | CCCATGTCGAGGTTTATTTACCC | 78 | A | unpublished data |
| POS_5863876 | ilMelCinx1.1 (GCF_905220565.1) | NC_059424.1 | 6245295 | T | A | NA |  | ACACTCTTTCCCTACACGACGCTCTTCCGATCT | AGTTTGTTTGAAGGAACCGC | GTGACTGGAGTTCAGACGTGTGCTCTTCCGATCT | GCGGTATGGTCAGTTGTTGG | 92 | B | unpublished data |
| POS_10484720 | ilMelCinx1.1 (GCF_905220565.1) | NC_059424.1 | 10739590 | A | T | NA | low | ACACTCTTTCCCTACACGACGCTCTTCCGATCT | GGGGTGTACGTCTTTCTGAA | GTGACTGGAGTTCAGACGTGTGCTCTTCCGATCT | TACCAAGAACACGCAATGCC | 137 | B | unpublished data |
| POS_13093607 | ilMelCinx1.1 (GCF_905220565.1) | NC_059424.1 | 13291635 | no SNP | no SNP | NA |  | ACACTCTTTCCCTACACGACGCTCTTCCGATCT | TGGCCGCTCAGTATTTGTTC | GTGACTGGAGTTCAGACGTGTGCTCTTCCGATCT | CATACGGATTGGGGAGACGA | 107 | B | unpublished data |
| s2291_R269 | ilMelCinx1.1 (GCF_905220565.1) | NC_059424.1 | 13761212 | T | C | NA | low | ACACTCTTTCCCTACACGACGCTCTTCCGATCT | CCTTCTCATAATGTTAAGTGCC | GTGACTGGAGTTCAGACGTGTGCTCTTCCGATCT | GCAAAGGATAGAACTGGAATGTC | 110 | A | unpublished data |

**Table S3**. Summary tables of quantile regression analysis for all WGCNA gene modules. Significant effects are showed in *italics*.

| Module | Quantile | Variable | Estimate | Std. error | LCI | UCI | P value |
| --- | --- | --- | --- | --- | --- | --- | --- |
|  |  | Intercept | -0.008 | 0.017 | -0.042 | 0.026 | 0.653 |
|  |  | Temperature | -0.021 | 0.018 | -0.057 | 0.014 | 0.227 |
| 1 | 0.25 | Population | -0.010 | 0.016 | -0.043 | 0.022 | 0.522 |
|  |  | Sex | 0.001 | 0.016 | -0.031 | 0.032 | 0.974 |
|  |  | Interaction | 0.041 | 0.077 | -0.113 | 0.196 | 0.593 |
|  |  | Intercept | 0.008 | 0.010 | -0.012 | 0.028 | 0.423 |
|  |  | Population | -0.004 | 0.011 | -0.026 | 0.018 | 0.707 |
| 1 | 0.5 | Sex | 0.012 | 0.007 | -0.002 | 0.025 | 0.095 |
|  |  | Interaction | 0.069 | 0.060 | -0.051 | 0.190 | 0.253 |
|  |  | Temperature | -0.016 | 0.012 | -0.041 | 0.009 | 0.209 |
|  |  | Intercept | 0.024 | 0.012 | -0.001 | 0.049 | 0.061 |
|  |  | Temperature | 0.006 | 0.019 | -0.033 | 0.045 | 0.756 |
| 1 | 0.75 | Population | 0.004 | 0.013 | -0.021 | 0.029 | 0.740 |
|  |  | Sex | 0.005 | 0.011 | -0.016 | 0.027 | 0.624 |
|  |  | Interaction | 0.063 | 0.045 | -0.028 | 0.153 | 0.172 |
|  |  | Intercept | -0.073 | 0.063 | -0.200 | 0.055 | 0.257 |
|  |  | Population | 0.088 | 0.068 | -0.049 | 0.225 | 0.203 |
| 2 | 0.5 | Sex | 0.003 | 0.021 | -0.039 | 0.046 | 0.875 |
|  |  | Interaction | 0.031 | 0.060 | -0.090 | 0.151 | 0.613 |
|  |  | Temperature | 0.031 | 0.053 | -0.075 | 0.138 | 0.556 |
|  |  | *Intercept* | *-0.107* | *0.004* | *-0.115* | *-0.098* | *< 0.001* |
|  |  | *Population* | *0.197* | *0.006* | *0.184* | *0.209* | *< 0.001* |
| 3 | 0.5 | Sex | -0.001 | 0.003 | -0.006 | 0.004 | 0.603 |
|  |  | Interaction | 0.008 | 0.010 | -0.012 | 0.028 | 0.430 |
|  |  | Temperature | 0.012 | 0.008 | -0.004 | 0.029 | 0.145 |
|  |  | *Intercept* | *0.094* | *0.006* | *0.081* | *0.106* | *< 0.001* |
|  |  | *Population* | *-0.196* | *0.007* | *-0.211* | *-0.182* | *< 0.001* |
| 4 | 0.5 | Sex | 0.000 | 0.002 | -0.005 | 0.005 | 0.947 |
|  |  | *Interaction* | *-0.019* | *0.008* | *-0.035* | *-0.002* | *0.026* |
|  |  | *Temperature* | *0.020* | *0.007* | *0.005* | *0.035* | *0.008* |
|  |  | *Temperature* | *-0.051* | *0.025* | *-0.100* | *-0.002* | *0.043* |
|  |  | *Intercept* | *0.051* | *0.009* | *0.032* | *0.070* | *< 0.001* |
| 5 | 0.5 | Population | 0.010 | 0.011 | -0.012 | 0.031 | 0.369 |
|  |  | Sex | -0.005 | 0.012 | -0.030 | 0.020 | 0.683 |
|  |  | *Interaction* | *-0.154* | *0.050* | *-0.254* | *-0.055* | *0.003* |
|  |  | *Temperature* | *-0.126* | *0.053* | *-0.233* | *-0.019* | *0.022* |
|  |  | Intercept | 0.032 | 0.032 | -0.033 | 0.097 | 0.334 |
| 6 | 0.5 | Population | 0.042 | 0.035 | -0.028 | 0.113 | 0.232 |
|  |  | Sex | 0.024 | 0.015 | -0.006 | 0.055 | 0.111 |
|  |  | Interaction | -0.041 | 0.067 | -0.177 | 0.094 | 0.540 |

|  |  | *Temperature* | *-0.181* | *0.025* | *-0.231* | *-0.131* | *< 0.001* |
| --- | --- | --- | --- | --- | --- | --- | --- |
|  |  | *Intercept* | *0.059* | *0.010* | *0.040* | *0.079* | *< 0.001* |
| 7 | 0.5 | Population | 0.001 | 0.014 | -0.026 | 0.028 | 0.943 |
|  |  | *Sex* | *0.030* | *0.009* | *0.011* | *0.050* | *0.002* |
|  |  | *Interaction* | *0.086* | *0.033* | *0.020* | *0.153* | *0.012* |
|  |  | Intercept | -0.012 | 0.021 | -0.054 | 0.030 | 0.560 |
|  |  | *Temperature* | *-0.162* | *0.020* | *-0.203* | *-0.122* | *< 0.001* |
| 8 | 0.25 | Population | -0.034 | 0.026 | -0.086 | 0.018 | 0.194 |
|  |  | Sex | 0.024 | 0.012 | 0.000 | 0.048 | 0.054 |
|  |  | *Interaction* | *0.125* | *0.049* | *0.027* | *0.222* | *0.013* |
|  |  | *Temperature* | *-0.146* | *0.025* | *-0.197* | *-0.096* | *< 0.001* |
|  |  | Intercept | 0.016 | 0.026 | -0.036 | 0.069 | 0.532 |
| 8 | 0.5 | Population | -0.030 | 0.025 | -0.080 | 0.020 | 0.239 |
|  |  | Sex | 0.018 | 0.022 | -0.026 | 0.062 | 0.411 |
|  |  | *Interaction* | *0.231* | *0.047* | *0.137* | *0.326* | *< 0.001* |
|  |  | *Intercept* | *0.057* | *0.023* | *0.012* | *0.102* | *0.015* |
|  |  | *Temperature* | *-0.097* | *0.041* | *-0.180* | *-0.015* | *0.022* |
| 8 | 0.75 | Population | -0.033 | 0.027 | -0.088 | 0.021 | 0.224 |
|  |  | Sex | 0.030 | 0.035 | -0.041 | 0.101 | 0.397 |
|  |  | *Interaction* | *0.209* | *0.048* | *0.112* | *0.305* | *< 0.001* |
|  |  | *Intercept* | *-0.074* | *0.014* | *-0.102* | *-0.045* | *< 0.001* |
|  |  | Temperature | 0.044 | 0.032 | -0.020 | 0.109 | 0.176 |
| 9 | 0.25 | Population | -0.021 | 0.015 | -0.051 | 0.008 | 0.146 |
|  |  | Sex | -0.005 | 0.011 | -0.027 | 0.017 | 0.644 |
|  |  | Interaction | 0.081 | 0.043 | -0.006 | 0.168 | 0.069 |
|  |  | *Intercept* | *-0.052* | *0.022* | *-0.096* | *-0.008* | *0.022* |
|  |  | *Temperature* | *0.080* | *0.035* | *0.011* | *0.150* | *0.024* |
| 9 | 0.5 | Population | -0.040 | 0.024 | -0.088 | 0.010 | 0.112 |
|  |  | Sex | -0.007 | 0.009 | -0.025 | 0.012 | 0.462 |
|  |  | Interaction | 0.065 | 0.048 | -0.031 | 0.161 | 0.178 |
|  |  | Intercept | -0.026 | 0.025 | -0.076 | 0.025 | 0.312 |
|  |  | Temperature | 0.073 | 0.035 | 0.004 | 0.143 | 0.040 |
| 9 | 0.75 | Population | -0.040 | 0.030 | -0.099 | 0.019 | 0.183 |
|  |  | Sex | 0.008 | 0.014 | -0.020 | 0.036 | 0.571 |
|  |  | *Interaction* | *0.166* | *0.052* | *0.060* | *0.271* | *0.003* |

|  |  | Intercept | -0.039 | 0.021 | -0.082 | 0.003 | 0.068 |
| --- | --- | --- | --- | --- | --- | --- | --- |
|  |  | Temperature | 0.060 | 0.041 | -0.023 | 0.144 | 0.151 |
| 10 | 0.25 | Population | 0.030 | 0.029 | -0.027 | 0.088 | 0.291 |
|  |  | Sex | -0.024 | 0.033 | -0.091 | 0.043 | 0.471 |
|  |  | *Interaction* | *-0.219* | *0.054* | *-0.327* | *-0.111* | *< 0.001* |
|  |  | Intercept | -0.009 | 0.022 | -0.055 | 0.036 | 0.676 |
|  |  | *Temperature* | *0.138* | *0.040* | *0.057* | *0.218* | *0.001* |
| 10 | 0.5 | Population | 0.023 | 0.027 | -0.030 | 0.077 | 0.387 |
|  |  | Sex | -0.004 | 0.017 | -0.038 | 0.029 | 0.788 |
|  |  | *Interaction* | *-0.232* | *0.061* | *-0.355* | *-0.109* | *< 0.001* |
|  |  | Intercept | 0.015 | 0.020 | -0.026 | 0.056 | 0.473 |
|  |  | *Temperature* | *0.137* | *0.022* | *0.093* | *0.180* | *< 0.001* |
| 10 | 0.75 | Population | 0.032 | 0.026 | -0.020 | 0.085 | 0.222 |
|  |  | Sex | -0.015 | 0.013 | -0.042 | 0.012 | 0.265 |
|  |  | Interaction | -0.087 | 0.054 | -0.195 | 0.022 | 0.116 |
|  |  | *Intercept* | *-0.087* | *0.012* | *-0.112* | *-0.062* | *< 0.001* |
|  |  | *Temperature* | *0.104* | *0.030* | *0.044* | *0.163* | *0.001* |
| 11 | 0.25 | Population | 0.016 | 0.015 | -0.014 | 0.045 | 0.291 |
|  |  | Sex | -0.015 | 0.023 | -0.062 | 0.032 | 0.524 |
|  |  | *Interaction* | *-0.077* | *0.030* | *-0.137* | *-0.017* | *0.013* |
|  |  | *Intercept* | *-0.053* | *0.009* | *-0.072* | *-0.035* | *< 0.001* |
|  |  | *Temperature* | *0.156* | *0.035* | *0.085* | *0.226* | *< 0.001* |
| 11 | 0.5 | Population | -0.003 | 0.010 | -0.023 | 0.016 | 0.728 |
|  |  | *Sex* | *-0.020* | *0.007* | *-0.034* | *-0.005* | *0.009* |
|  |  | *Interaction* | *-0.097* | *0.045* | *-0.188* | *-0.006* | *0.037* |
|  |  | *Intercept* | *-0.050* | *0.008* | *-0.066* | *-0.034* | *< 0.001* |
|  |  | *Temperature* | *0.253* | *0.023* | *0.207* | *0.298* | *< 0.001* |
| 11 | 0.75 | Population | -0.002 | 0.009 | -0.021 | 0.017 | 0.841 |
|  |  | Sex | -0.006 | 0.008 | -0.022 | 0.010 | 0.462 |
|  |  | Interaction | -0.076 | 0.061 | -0.198 | 0.046 | 0.215 |
